# Comparative evaluation of five nutrition-inflammation indices for predicting 28-day ICU mortality in critically ill patients with bone infections: a dual-cohort study with external validation and interpretable machine learning

**DOI:** 10.3389/fnut.2026.1949102

**Published:** 2026-09-16

**Authors:** Zishuo Xi, Jialin He, Mingyang Wang, Zhongyi Chen, Guiyang Zi, Wenkai Su, Bin Yue

**Affiliations:** Department of Bone Oncology, The Affiliated Hospital of Qingdao University, Qingdao, China

**Keywords:** bone infections, external validation, HAlP, ICU mortality, machine learning, nutrition-inflammation indices, predictive model, SHAP

## Abstract

**Background:**

Malnutrition and systemic inflammation are highly prevalent among critically ill patients with bone infections and may independently predict adverse outcomes. Five composite nutrition-inflammation indices—the modified Nutrition Risk in the Critically Ill (mNUTRIC) score, Prognostic Nutritional Index (PNI), Geriatric Nutritional Risk Index (GNRI), Index of Nutritional Assessment (INA), and Hemoglobin-Albumin-Lymphocyte-Platelet (HALP) score—have been proposed as prognostic tools; however, their comparative performance in the bone infection intensive care unit (ICU) population has not been systematically defined.

**Material and method:**

A dual-cohort retrospective study design was employed. The internal derivation cohort comprised ICU patients with bone infections from the Medical Information Mart for Intensive Care IV (MIMIC-IV) database (*n* = 917; 28-day ICU mortality 12.1%), and the external validation cohort was drawn from the Affiliated Hospital of Qingdao University (*n* = 256; 28-day mortality 16.0%). All five indices were calculated from laboratory and clinical data collected within 24 h of ICU admission. Multivariable logistic regression (three hierarchical adjustment models), restricted cubic spline (RCS) analysis, and subgroup analyses were performed to examine the independent associations of each index with 28-day ICU mortality. For predictive model development, a dual LASSO-Boruta feature selection strategy was applied to the derivation cohort, yielding 14 core features (events-per-variable ratio [EPV] = 7.9). Five machine learning (ML) models — logistic regression (LR), random forest (RF), XGBoost, LightGBM, and support vector machine (SVM) — were trained with synthetic minority over-sampling technique (SMOTE) and five-fold stratified cross-validation. Discriminative performance was assessed by the area under the receiver operating characteristic curve (AUROC). The optimal model was further evaluated using SHapley Additive exPlanations (SHAP) values computed directly for the final LightGBM model with external stability validation, calibration curves, and decision curve analysis (DCA). A fully nested cross-validation with feature selection repeated within each training fold confirmed the robustness of the external performance.

**Result:**

A total of 917 ICU patients with bone infections were included in the derivation cohort. In the fully adjusted model, HALP lowest tertile (T1, ≤ 171.11; odds ratio [OR] = 3.41, 95% confidence interval [CI] 2.13–5.46, *p* < 0.001) and low PNI (< 45; OR = 2.48, 95% CI 1.09–5.66, *p* = 0.031) remained independently associated with 28-day ICU mortality, whereas the associations of mNUTRIC, GNRI, and INA were attenuated to non-significance after full adjustment. RCS analysis confirmed a significant nonlinear dose-response relationship for HALP (*p*-nonlinearity < 0.001). In both cohorts, PNI (derivation AUROC 0.761; external 0.742) and HALP (0.745; 0.713) demonstrated the highest discriminative performance among the five indices, while GNRI lost discrimination owing to a near-universal at-risk classification (94.9% of the derivation cohort). Subgroup analysis showed the HALP–mortality association was consistent across all clinical strata (all interaction *p* > 0.27). Among ML models, LightGBM achieved the highest external AUROC (0.956, 95% CI 0.921–0.985). SHAP analysis of the final LightGBM model identified APS III, acute kidney injury (AKI), and arterial oxygen tension (PO_2_) as the most important predictors, with the ranking highly stable in the external validation cohort. DCA demonstrated superior net clinical benefit for the ML model over any single index across clinically relevant threshold probabilities.

**Conclusion:**

Among five nutrition-inflammation indices evaluated in critically ill patients with bone infections, HALP and PNI were the leading independent predictors of 28-day ICU mortality, with HALP showing a robust, strongly nonlinear threshold effect that was consistent across all subgroups and adjustment models. In contrast to its dominant role in cardiothoracic populations, GNRI lost discriminative value here because nearly all patients were classified as at-risk. An interpretable ML model integrating 14 routinely available clinical features achieved excellent external discrimination (AUROC = 0.956) and clinical utility, supporting its potential as a decision-support tool for early risk stratification in this high-risk population.

## Introduction

Bone infections, encompassing bone infections and septic arthritis, are progressive inflammatory destructive processes of bone and joint tissues caused by pyogenic microorganisms, most commonly Staphylococcus aureus, and remain a substantial and growing clinical burden worldwide (1, 2). Population-based studies report a rising incidence driven by an aging population and the increasing prevalence of diabetes mellitus, peripheral vascular disease, and orthopedic implant procedures (1, 3, 4). Although bone infections are frequently regarded as localized skeletal or joint infections, severe cases—including vertebral bone infections, complicated diabetic foot infections, septic arthritis, and haematogenous disease with sepsis—carry considerable morbidity and mortality and often require admission to the intensive care unit (ICU) (5, 6). Reported in-hospital and long-term mortality in these severe presentations ranges widely but can exceed 15%−20%, and recent ICU-based analyses have begun to define the prognostic determinants of critically ill patients with bone infections (7–9).

The pathophysiology of bone infections is characterized by a self-perpetuating cycle of infection, immune activation, and tissue catabolism (10, 11). S. aureus subverts both innate and adaptive immunity, forms biofilms and intracellular reservoirs within bone, and drives sustained systemic inflammation (11, 12). In septic arthritis, similar immune-inflammatory cascades occur within the synovial space, leading to cartilage degradation and systemic inflammatory responses. This chronic inflammatory state accelerates protein-energy wasting, and malnutrition in turn impairs neutrophil function, lymphocyte proliferation, and antibody responses, creating a vicious circle of immune dysfunction and impaired host defense (13, 14). Experimental evidence further shows that inflammatory stimuli remodel the bone marrow and directly compromise bone health in the malnourished host (15). Consistent with this biology, malnutrition has been repeatedly associated with periprosthetic joint infection, surgical-site infection, and adverse outcomes across orthopedic and infectious diseases (16). Objective, inexpensive markers that capture the combined nutritional and inflammatory state of the host are therefore particularly attractive for risk stratification in bone infections.

Several composite nutrition-inflammation indices, each computable from routine laboratory and clinical data, have been developed and validated in critically ill and surgical populations. The modified Nutrition Risk in the Critically Ill (mNUTRIC) score integrates age, illness severity, organ dysfunction, and comorbidity to identify patients most likely to benefit from nutritional therapy and predicts 28-day mortality across ICU cohorts (17–20). The Prognostic Nutritional Index (PNI), derived from serum albumin and lymphocyte count, predicts mortality in sepsis, ventilator-associated pneumonia, and diabetic foot disease (21–23). The Geriatric Nutritional Risk Index (GNRI), based on albumin and the ratio of actual to ideal body weight, predicts short-term mortality in older and septic patients (24–26). The Index of Nutritional Assessment (INA) combines hypoalbuminaemia and lymphopenia into a simple four-grade classification (27). The Hemoglobin-Albumin-Lymphocyte-Platelet (HALP) score, originally proposed in gastric cancer, integrates four hematological and nutritional parameters and has since shown broad prognostic value across malignancies, in the general population, and in sepsis (28–32).

Despite this growing body of literature, no study has systematically compared these five nutrition-inflammation indices head-to-head in critically ill patients with bone infections—a population defined by the intersection of chronic infection, systemic inflammation, and protein-energy malnutrition in which such indices are biologically most plausible. Prior ICU studies of bone infections have examined individual laboratory markers rather than integrated nutrition-inflammation indices (7–9), and the relative and independent prognostic performance of mNUTRIC, PNI, GNRI, INA, and HALP in this setting is unknown. Furthermore, whether these indices add value beyond, or can be integrated with, modern interpretable machine learning (ML) for early mortality prediction has not been evaluated.

To address these gaps, we conducted a dual-cohort study that (1) systematically compared mNUTRIC, PNI, GNRI, INA, and HALP for their association with and discrimination of 28-day ICU mortality in patients with bone infections, using the MIMIC-IV database as the derivation cohort and an independent cohort from the Affiliated Hospital of Qingdao University for external validation; (2) characterized the shape of each dose-response relationship using restricted cubic splines and tested the consistency of the leading index across clinical subgroups; and (3) developed and externally validated an interpretable ML model, using SHAP analysis, calibration, and decision-curve analysis to assess its predictive mechanisms and clinical utility. To our knowledge, this is the first systematic head-to-head comparison of five nutrition-inflammation indices for 28-day ICU mortality in critically ill patients with bone infections, combined with external validation and interpretable ML.

## Materials and methods

### Study design and data sources

For both cohorts (with the internal derivation cohort drawn from the Medical Information Mart for Intensive Care IV [MIMIC-IV] database, version 2.2, which comprises de-identified records of patients admitted to the Beth Israel Deaconess Medical Center between 2008 and 2019, pre-dating the COVID-19 pandemic), adult patients (aged ≥18 years) with a diagnosis of bone infections who were admitted to the ICU were identified using the relevant International Classification of Diseases (ICD-9 and ICD-10) diagnosis codes. Bone infections were defined to include both osteomyelitis (ICD-9: 730.xx; ICD-10: M86.xx) and septic arthritis/pyogenic arthritis (ICD-9: 711.xx; ICD-10: M00.x, M01.x, M02.x, etc.), as well as other specified infections of bone and joint (e.g., ICD-10 M86.8, M86.9). Consistent with the pre-2020 coverage period, none of the ICD-9/ICD-10 diagnosis codes used for cohort identification corresponds to COVID-19 (ICD-10 U07.1); consequently, COVID-19-related confounding of lymphocyte counts, inflammatory dynamics, and nutritional parameters does not affect this cohort. A full list of included codes is provided in the Supplementary Materials. Inclusion criteria were: (1) age ≥18 years; (2) a documented diagnosis of bone infection according to the above codes; (3) first ICU admission; and (4) availability of the laboratory and clinical components required to compute the nutrition-inflammation indices within 24 h of ICU admission. Exclusion criteria were: (1) missing primary outcome data; and (2) repeat ICU admissions (only the first ICU admission per patient was retained).

### Study population

For both cohorts, adult patients (aged ≥18 years) with a diagnosis of bone infections who were admitted to the ICU were identified using the relevant International Classification of Diseases (ICD-9 and ICD-10) diagnosis codes. Inclusion criteria were: (1) age ≥18 years; (2) a documented diagnosis of bone infections; (3) first ICU admission; and (4) availability of the laboratory and clinical components required to compute the nutrition-inflammation indices within 24 h of ICU admission. Exclusion criteria were: (1) missing primary outcome data; and (2) repeat ICU admissions (only the first ICU admission per patient was retained). Pregnancy and trauma were not applied as specific exclusion criteria. No pregnancy-related diagnostic codes (ICD-9 630–679; ICD-10 O00–O9A) were identified among the included patients, consistent with the advanced age structure of the cohort. Patients with post-traumatic or post-surgical bone infections were eligible, as these conditions represent integral etiologies of bone infection in the critically ill population. After applying these criteria, 917 patients were included in the MIMIC-IV derivation cohort and 256 patients in the external validation cohort.

### Variable extraction

Data extraction for the derivation cohort was performed using structured query language (SQL) against the MIMIC-IV relational database. For each patient, the first recorded value of each laboratory and vital-sign parameter within 24 h of ICU admission was extracted, including complete blood count (hemoglobin, platelet count, white blood cell count, and lymphocyte and neutrophil counts and percentages), serum albumin, creatinine, blood urea nitrogen, lactate, chloride, glucose, fibrinogen, red blood cell count, red cell distribution width (RDW), and arterial blood gas parameters (PO_2_, peripheral oxygen saturation [SpO_2_]). Demographic variables (age, sex, height, and weight), comorbidities (hypertension, diabetes mellitus, chronic kidney disease, heart failure, chronic obstructive pulmonary disease, and acute kidney injury [AKI]), the Charlson Comorbidity Index, illness-severity scores (SOFA, APACHE II, APS III, SAPS II, OASIS, SIRS, and the Glasgow Coma Scale), and interventions (mechanical ventilation, vasopressor use, and antibiotic administration) were also extracted. A pre-specified missing-data threshold of 30% was applied, consistent with common practice in large critical-care database studies and with methodological evidence that multiple imputation performs acceptably under a missing-at-random assumption even at moderate-to-substantial fractions of missing data. In the final analytic cohort, all extracted variables had less than 30% missingness (range 0.1%−29.1%), so no variable was excluded by this criterion. Among the variables presented in the baseline table (Table 1), only white blood cell count (0.3%) and lactate (4.1%) had any missing values in the derivation cohort, and all baseline variables were complete in the external validation cohort; critically, all components of the five nutrition–inflammation indices (albumin, lymphocyte count, hemoglobin, platelet count, body weight, height) and all covariates used in the logistic regression models had 0% missingness in both cohorts, so the primary regression results are based entirely on observed data. Fibrinogen (29.1% missing in the derivation cohort) was the only variable approaching the threshold; it is not a component of any index or a covariate in the regression models, and enters the analysis solely as one of the 14 features of the machine-learning model. A sensitivity analysis excluding fibrinogen from the machine-learning feature set yielded an essentially unchanged external AUROC (0.956 with fibrinogen vs. 0.956 without), confirming that the missing-data handling did not materially influence the conclusions. A complete missingness summary is provided in Supplementary Table S4. Variables with less than 30% missingness were imputed by multiple imputation using the mice package in *R* (predictive mean matching for continuous variables; logistic or polytomous regression for categorical variables), generating *m* = 5 imputed datasets that were pooled using Rubin's rules.

**Table 1:** Baseline characteristics of study cohorts.

Variable	Non-survivors (*n* = 111)	Survivors (*n* = 806)	*P*-value	External validation (*n* = 256)
Demographics				
Age, years	75.0 (68.0–80.5)	71.0 (63.0–78.0)	< 0.001	71.0 (63.0–79.0)
Male, *n* (%)	53 (47.7%)	459 (56.9%)	0.084	136 (53.1%)
Weight, kg	80.0 (65.0–97.9)	84.0 (71.5–99.2)	0.104	83.2 (69.8–98.2)
Height, cm	165.0 (157.0–173.0)	170.0 (163.0–178.0)	0.018	168.0 (160.0–175.0)
Comorbidities				
Hypertension, *n* (%)	35 (31.5%)	393 (48.8%)	< 0.001	125 (48.8%)
Diabetes mellitus, *n* (%)	35 (31.5%)	283 (35.1%)	0.524	80 (31.2%)
Chronic kidney disease, *n* (%)	43 (38.7%)	162 (20.1%)	< 0.001	53 (20.7%)
Heart failure, *n* (%)	52 (46.8%)	229 (28.4%)	< 0.001	65 (25.4%)
COPD, *n* (%)	32 (28.8%)	100 (12.4%)	< 0.001	35 (13.7%)
Acute kidney injury, *n* (%)	90 (81.1%)	306 (38.0%)	< 0.001	116 (45.3%)
Charlson Comorbidity Index	7.0 (6.0–9.0)	5.0 (3.0–7.0)	< 0.001	5.0 (3.0–7.0)
Severity scores				
SOFA score	8.0 (5.5–10.0)	5.0 (3.0–7.0)	< 0.001	5.5 (4.0–8.0)
APACHE II score	24.0 (19.0–29.0)	19.0 (15.0–23.0)	< 0.001	20.0 (16.0–24.0)
APS III score	61.0 (49.5–74.0)	39.0 (30.0–52.0)	< 0.001	42.0 (32.0–58.0)
SAPS II score	48.0 (40.0–58.0)	36.0 (30.0–45.0)	< 0.001	39.0 (31.0–48.0)
OASIS score	38.0 (31.5–43.0)	31.0 (26.0–37.0)	< 0.001	32.0 (27.0–38.0)
SIRS score	3.0 (2.0–3.0)	3.0 (2.0–3.0)	0.010	3.0 (2.0–3.0)
Glasgow Coma Scale	15.0 (13.0–15.0)	15.0 (14.0–15.0)	0.001	15.0 (14.0–15.0)
Laboratory values				
Albumin, g/dl	2.6 (2.2–3.1)	3.1 (2.8–3.4)	< 0.001	3.0 (2.6–3.3)
Hemoglobin, g/dl	8.9 (7.8–10.3)	9.3 (8.1–10.8)	0.043	9.1 (7.8–10.3)
Platelet, 103/ul	161.0 (101.0–281.0)	153.0 (115.0–209.8)	0.311	154.5 (115.0–216.0)
WBC, 103/ul	13.2 (9.4–18.1)	11.9 (8.6–16.5)	0.067	13.1 (9.0–18.2)
Lymphocytes, 10^3^/ul	0.7 (0.5–1.2)	1.5 (1.0–2.3)	< 0.001	1.4 (0.8–2.2)
Neutrophils, 10^3^/ul	10.4 (7.5–15.9)	8.9 (6.1–12.9)	0.001	9.8 (6.4–14.5)
Creatinine, mg/dl	1.4 (1.0–2.8)	0.9 (0.7–1.3)	< 0.001	1.0 (0.7–1.5)
Lactate, mmol/L	1.9 (1.4–2.8)	1.8 (1.3–2.6)	0.287	1.9 (1.4–2.7)
BUN, mg/dl	35.0 (24.0–57.0)	17.0 (13.0–25.8)	< 0.001	18.0 (13.0–30.0)
Interventions				
Mechanical ventilation, *n* (%)	103 (92.8%)	752 (93.3%)	1.000	242 (94.5%)
Vasopressor use, *n* (%)	101 (91.0%)	671 (83.3%)	0.050	218 (85.2%)
Antibiotic use, *n* (%)	110 (99.1%)	732 (90.8%)	< 0.001	235 (91.8%)
Nutrition-inflammation indices				
mNUTRIC score	7.0 (5.0–8.0)	5.0 (4.0–6.0)	< 0.001	4.0 (3.0–5.2)
PNI	30.8 (25.5–36.9)	39.1 (34.3–43.8)	< 0.001	37.9 (32.4–42.5)
GNRI	80.4 (73.2–87.9)	87.9 (82.5–92.3)	< 0.001	86.4 (80.4–90.8)
HALP	102.8 (49.3–213.9)	292.2 (149.0–470.3)	< 0.001	246.2 (120.4–411.9)
mNUTRIC high risk, *n* (%)	100 (90.1%)	487 (60.4%)	< 0.001	177 (69.1%)
PNI < 45 (low), *n* (%)	104 (93.7%)	645 (80.0%)	< 0.001	222 (86.7%)
GNRI < 98 (at risk), *n* (%)	109 (98.2%)	761 (94.4%)	0.108	246 (96.1%)
INA grade 3–4 (high risk), *n* (%)	105 (94.6%)	726 (90.1%)	0.175	236 (92.2%)
HALP T1 (lowest tertile), *n* (%)	74 (66.7%)	232 (28.8%)	< 0.001	93 (36.3%)

Continuous variables are presented as median (interquartile range); categorical variables as *n* (%). *P*–values are from the Mann-Whitney U test (continuous) or the chi-squared test (categorical) comparing non-survivors vs. survivors in the MIMIC-IV derivation cohort. The external validation column (*n* = 256) reports the overall cohort. WBC, white blood cell count; BUN, blood urea nitrogen; SOFA, Sequential Organ Failure Assessment; APACHE II, Acute Physiology and Chronic Health Evaluation II; APS III, Acute Physiology Score III; SAPS II, Simplified Acute Physiology Score II; OASIS, Oxford Acute Severity of Illness Score; SIRS, systemic inflammatory response syndrome; COPD, chronic obstructive pulmonary disease; mNUTRIC = modified Nutrition Risk in Critically Ill; PNI, Prognostic Nutritional Index; GNRI, Geriatric Nutritional Risk Index; INA, Index of Nutritional Assessment; HALP, hemoglobin, albumin, lymphocyte, and platelet score.

### Outcome definition

The primary outcome was 28-day ICU mortality, defined as death occurring within 28 days of ICU admission.

### Definition and calculation of nutrition-inflammation indices

All five nutrition-inflammation indices were calculated from laboratory and clinical data collected within 24 h of ICU admission. The absolute lymphocyte count in cells/mm^3^ was derived from the reported absolute lymphocyte count where available. The mNUTRIC score was calculated using five components in the MIMIC-IV derivation cohort: age, APACHE II score, SOFA score, number of comorbidities, and days from hospital admission to ICU admission, with a high-risk threshold of ≥5. For the external validation cohort, a four-component mNUTRIC score was applied (excluding the days-to-ICU component, which was unavailable), with a high-risk threshold of ≥4, adjusted from the five-component threshold (17). PNI was calculated as PNI = 10 × albumin (g/dl) + 0.005 × lymphocyte count (cells/mm^3^), with a low-PNI threshold of < 45 (21). GNRI was calculated as GNRI = 1.489 × albumin (g/L) + 41.7 × (body weight/ideal body weight [IBW]), where IBW was estimated using the Devine formula and the body weight-to-IBW ratio was capped at 1.0; a GNRI < 98 defined nutritional risk (24). INA stratified patients into four grades by the combination of serum albumin and lymphocyte count: grade 1 (albumin >3.5 g/dl and lymphocytes >1,500 cells/mm^3^), grade 2 (albumin >3.5 g/dl and lymphocytes ≤ 1,500 cells/mm^3^), grade 3 (albumin ≤ 3.5 g/dl and lymphocytes >1,500 cells/mm^3^), and grade 4 (albumin ≤ 3.5 g/dl and lymphocytes ≤ 1,500 cells/mm^3^); grades 3–4 were classified as high risk (27). The HALP score was calculated as HALP = (hemoglobin × albumin × lymphocyte count)/platelet count. Tertile cut-off values were derived from the MIMIC-IV derivation cohort and applied to both cohorts (T1 ≤ 171.11; T2 ≤ 380.84), with the lowest tertile (T1) defined as high risk (28).

### Subgroup analysis

To assess the consistency of the association between the leading nutrition-inflammation index and 28-day ICU mortality across clinically relevant strata, subgroup analyses were performed according to age (< 70 vs. ≥70 years), sex, diabetes mellitus, AKI, chronic kidney disease, and heart failure. Within each subgroup, logistic regression adjusted for age, sex, and APACHE II score (excluding the stratifying variable) was used to estimate the odds ratio (OR). Effect modification was tested by including a multiplicative interaction term between the index and the subgroup variable, with the interaction *p*-value reported.

### Feature selection and machine learning model development

Feature selection was conducted in two sequential stages applied to the derivation cohort. In the first stage, LASSO (L1-penalized logistic regression) was performed with the regularization strength selected by cross-validation. In the second stage, the Boruta algorithm (150 iterations) was applied as an all-relevant wrapper method built on a random forest classifier. Candidate features comprised the 71 clinical, laboratory, and nutrition-inflammation variables shared between the two cohorts; ICU length of stay was excluded *a priori* to prevent outcome leakage. Features selected by both LASSO and Boruta (their intersection) were retained, yielding 14 final features and an events-per-variable ratio (EPV) of 7.9. Because this selection was performed on the full derivation cohort, the cross-validated performance estimates are not fully independent of the selection step; we therefore additionally performed a fully nested sensitivity analysis in which the entire LASSO–Boruta selection procedure was repeated independently within each training fold of the five-fold cross-validation, followed by SMOTE and model fitting within that fold (Supplementary Table S8). Five ML models—logistic regression (LR), random forest (RF), XGBoost, LightGBM, and support vector machine (SVM)—were trained on the derivation cohort. These five algorithms were selected *a priori* to span the three principal algorithmic families used in clinical prediction modeling, so that the comparison is informative about model class rather than about any single algorithm, thereby ensuring that our findings are generalizable across algorithmic paradigms rather than being an artifact of a particular implementation. The rationale for each specific model is as follows: (1) logistic regression was included as a linear, fully interpretable baseline that remains the reference standard for clinical risk prediction, providing direct effect-size quantification and serving as a benchmark against which more complex models must justify their added value; (2) support vector machine with a radial basis function kernel was selected to capture smooth nonlinear decision boundaries without relying on explicit feature engineering, offering robustness against overfitting in structured clinical data through its margin-maximization objective; (3) random forest was included as a bagging ensemble that builds multiple decorrelated decision trees and averages their predictions, handling nonlinearity and feature interactions natively with reduced variance; (4) XGBoost was selected as one of the most widely adopted gradient-boosting implementations, characterized by level-wise tree growth, built-in regularization, and demonstrated state-of-the-art performance across numerous critical-care prediction tasks; and (5) LightGBM was additionally retained alongside XGBoost because it employs a complementary leaf-wise tree-growth strategy with distinct regularization and computational-efficiency characteristics, allowing us to evaluate whether these algorithmic distinctions translate into meaningful performance differences in our specific clinical context. This panel is consistent with recent peer-reviewed ICU prediction studies and balances interpretability, nonlinear pattern capture, reproducibility, and computational feasibility. To address class imbalance (event rate 12.1%), the synthetic minority over-sampling technique (SMOTE) was applied within each training fold of a five-fold stratified cross-validation. Model discrimination was quantified by the AUROC, the area under the precision-recall curve (AUPRC), and the Brier score, and every model was then evaluated on the independent external validation cohort.

### Model Interpretability analysis

To enhance the clinical interpretability of the ML models, SHapley Additive exPlanations (SHAP) analysis was performed. SHAP values were computed directly for the final LightGBM model using the exact TreeExplainer algorithm, both in the derivation cohort (*n* = 917) and, as an independent stability validation, in the external validation cohort (*n* = 256). Global feature importance was summarized as the mean absolute SHAP value, and beeswarm and dependence representations were used to display the direction and magnitude of each feature's contribution to the predicted probability of 28-day ICU mortality. Stability of the SHAP ranking between the two cohorts was assessed using Spearman's rank correlation.

### Statistical analysis

Statistical analyses were performed using R and Python, with R applied exclusively for multiple imputation. Multiple imputation was performed in R (version 4.x) using the mice package with predictive mean matching (*m* = 5 imputed datasets), and the completed datasets were exported for subsequent analyses. All remaining analyses were conducted in Python 3.11 (scikit-learn, scipy, statsmodels, xgboost, lightgbm, and shap). Continuous variables are presented as median (interquartile range [IQR]) and compared between survivors and non-survivors using the Mann–Whitney *U*–test. Categorical variables are presented as frequency (percentage) and compared using Pearson's chi-squared test or Fisher's exact test, as appropriate. Multivariable logistic regression was performed for each index across three sequential adjustment models: Model 1 (unadjusted); Model 2 (adjusted for age, sex, and APACHE II score); and Model 3 (fully adjusted: age, sex, APACHE II score, SOFA score, AKI, Charlson Comorbidity Index, mechanical ventilation, and vasopressor use). Results are reported as ORs with 95% confidence intervals (CIs). ROC analysis was performed for each index, with the AUROC and its 95% CI estimated using 1,000 stratified bootstrap iterations; protective indices were sign-inverted so that higher values consistently denoted higher risk. In addition, the optimal cut-off value of each index was determined by maximizing Youden's index (*J* = sensitivity + specificity − 1) in the derivation cohort, and the corresponding sensitivity, specificity, positive predictive value, negative predictive value, and accuracy were calculated; the derivation-cohort cut-offs were then applied unchanged to the external validation cohort (Table 2). Nonlinear relationships between continuous index values and 28-day ICU mortality were modeled using RCS analysis with four knots placed at the 5th, 35th, 65th, and 95th percentiles of each index distribution (33), with tests for overall and nonlinear associations. A two-sided *p*-value < 0.05 was considered statistically significant.

**Table 2:** Optimal cut-off values (Youden's index) and predictive metrics of the five nutrition–inflammation indices for 28-day ICU mortality.

Index	Cohort	AUROC	Cut-off	Youden J	Sens. (95% CI)	Spec. (95% CI)	PPV	NPV	Acc.
mNUTRIC	Derivation (MIMIC-IV)	0.723	≥ 6	0.359	0.784 (0.707–0.856)	0.576 (0.542–0.609)	0.203	0.951	0.601
mNUTRIC	External validation	0.731	≥ 6	-	0.683 (0.537–0.825)	0.753 (0.694–0.810)	0.346	0.926	0.742
PNI	Derivation (MIMIC-IV)	0.761	≤ 35.15	0.406	0.694 (0.602–0.779)	0.712 (0.683–0.743)	0.249	0.944	0.710
PNI	External validation	0.742	≤ 35.15	–	0.756 (0.605–0.886)	0.688 (0.630–0.750)	0.316	0.937	0.699
GNRI	Derivation (MIMIC-IV)	0.689	≤ 81.90	0.322	0.568 (0.474–0.664)	0.754 (0.724–0.782)	0.241	0.927	0.732
GNRI	External validation	0.704	≤ 81.90	–	0.610 (0.456–0.758)	0.721 (0.665–0.777)	0.294	0.906	0.703
INA	Derivation (MIMIC-IV)	0.671	≥4	0.35	0.793 (0.717–0.858)	0.557 (0.524–0.592)	0.198	0.951	0.586
INA	External validation	0.619	≥4	–	0.683 (0.529–0.821)	0.553 (0.491–0.615)	0.226	0.902	0.574
HALP	Derivation (MIMIC-IV)	0.745	≤ 191.91	0.406	0.730 (0.646–0.811)	0.676 (0.644–0.707)	0.237	0.948	0.683
HALP	External validation	0.713	≤ 191.91	–	0.732 (0.595–0.857)	0.651 (0.582–0.713)	0.286	0.927	0.664

Optimal cut-offs were derived by maximizing Youden's index in the derivation cohort and were applied unchanged to the external validation cohort. AUROC values are as reported in Table 4 of the manuscript (1,000 stratified bootstrap iterations). 95% confidence intervals for sensitivity and specificity were estimated with 1,000 stratified bootstrap iterations. Protective indices (PNI, GNRI, HALP) were sign-inverted for the ROC analysis so that higher values denote higher risk; cut-offs are reported on the original scale. AUROC, area under the receiver operating characteristic curve; PPV, positive predictive value; NPV, negative predictive value.

### Multicollinearity and model specification

To address potential multicollinearity among covariates in the fully adjusted logistic regression model (Model 3), variance inflation factor (VIF) was computed for all covariates, with VIF ≥ 5 prespecified as the threshold for problematic collinearity. The linearity-in-the-logit assumption for continuous covariates was assessed using the Box–Tidwell procedure by incorporating x·ln(x) interaction terms into the model. Furthermore, to avoid over-adjustment for covariates embedded within the indices themselves, component-aware sensitivity models were fitted: for mNUTRIC, which incorporates age, APACHE II, SOFA, and comorbidity count in its calculation, a model adjusting only for non-embedded covariates (sex, acute kidney injury, mechanical ventilation, and vasopressor use) was constructed; for all five indices, additional sensitivity models were fitted excluding SOFA, excluding APACHE II, excluding acute kidney injury, and excluding both SOFA and acute kidney injury, respectively.

## Result

### Baseline characteristics of study participants

The derivation cohort comprised 917 critically ill patients with bone infections (median age 72 years [IQR 63–78]; 55.8% male; 28-day ICU mortality 12.1% [111/917]). The external validation cohort comprised 256 patients (median age 71 years [IQR 63–79]; 53.1% male; 28-day mortality 16.0% [41/256]). Detailed baseline characteristics are presented in Table 1 and Supplementary Table S1. Within the derivation cohort, non-survivors were significantly older than survivors (median 75 vs. 71 years, *p* < 0.001) and had markedly higher illness-severity scores (SOFA 8 vs. 5; APACHE II 24 vs. 19; APS III 61 vs. 39; all *p* < 0.001) and a substantially higher incidence of AKI (81.1% vs. 38.0%, *p* < 0.001). Non-survivors also had significantly lower serum albumin (median 2.6 vs. 3.1 g/dl, *p* < 0.001), lower lymphocyte counts (0.7 vs. 1.5 × 10^3^/μl, *p* < 0.001), higher blood urea nitrogen (35 vs. 17 mg/dl, *p* < 0.001), and a higher Charlson Comorbidity Index (7 vs. 5, *p* < 0.001). Antibiotics were administered to the large majority of patients in both survival groups (99.1% of non-survivors and 90.8% of survivors). The external validation cohort had a higher baseline mortality rate (16.0 vs. 12.1%), and the pattern of between-group differences was broadly consistent with that observed in the derivation cohort (Table 1, Supplementary Table S1).

### Distribution of nutrition-inflammation indices

The five indices differed markedly in the proportion of patients they classified as being at nutritional or inflammatory risk. In the derivation cohort, 94.9% of patients were classified as at-risk by GNRI (< 98), 90.6% as high-risk by INA (grade 3–4), 81.7% as having low PNI (< 45), and 64.0% as high-risk by mNUTRIC (≥5), whereas by construction 33.4% fell within the lowest HALP tertile (T1). The Spearman correlation structure among the five indices is shown in Figure 1; PNI and GNRI were strongly positively correlated, consistent with their shared dependence on serum albumin, while HALP contributed partially independent information through its hemoglobin and platelet components. The distributions of each index stratified by survival status are shown in Supplementary Figure S1. To assess whether the use of different mNUTRIC variants across cohorts affected the comparability of the results, we performed a harmonized sensitivity analysis in which the 4-item mNUTRIC score (threshold ≥4) was recomputed in the derivation cohort, enabling a like-for-like comparison with the external validation cohort. Under this harmonized definition, the proportion of patients classified as high-risk in the derivation cohort was essentially unchanged (66.7 vs. 66.6% for the 5-item version), as was the discrimination (AUROC 0.733 vs. 0.734) and the fully adjusted association with 28-day ICU mortality (OR 1.67, 95% CI 0.75–3.73, *p* = 0.212 vs. OR 1.72, 95% CI 0.77–3.83, *p* = 0.188 for the 5-item version). When the harmonized binary mNUTRIC feature (4-item, ≥4) was used to retrain the machine-learning model, the external-validation AUROC remained essentially identical (0.957 vs. 0.956). These findings confirm that the definition shift between the 5-item and 4-item mNUTRIC variants does not materially influence the study conclusions (Supplementary Table S9).

**Figure 1:**
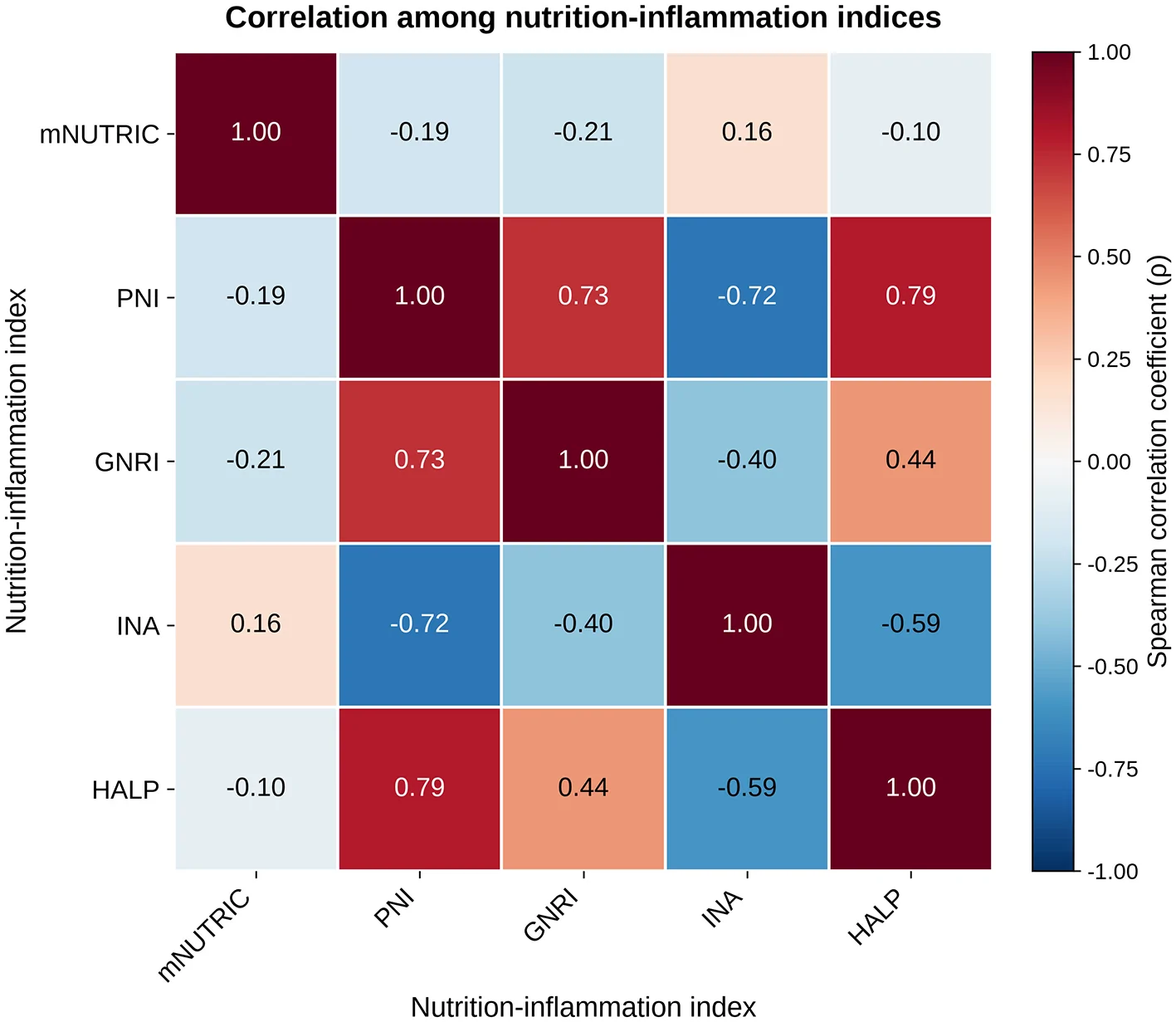
Spearman correlation matrix of five nutrition-inflammation indices in the MIMIC-IV derivation cohort. Color intensity represents the magnitude of Spearman's rank correlation coefficient (ρ). Positive correlations are shown in warm colors and negative correlations in cool colors. PNI and GNRI were strongly positively correlated owing to their shared dependence on serum albumin, whereas HALP contributed partially independent information through its hemoglobin and platelet components. mNUTRIC, modified Nutrition Risk in Critically Ill; PNI, Prognostic Nutritional Index; GNRI, Geriatric Nutritional Risk Index; INA, Index of Nutritional Assessment; HALP, hemoglobin, albumin, lymphocyte, and platelet score.

### Multivariable logistic regression analysis of nutrition-inflammation indices and 28-day ICU mortality

In the unadjusted model (Model 1), four of the five indices were significantly associated with 28-day ICU mortality: mNUTRIC ≥5 (OR = 5.95, 95% CI 3.14–11.28, *p* < 0.001), PNI < 45 (OR = 3.71, 95% CI 1.69–8.13, *p* = 0.001), INA grade 3–4 (OR = 1.93, 95% CI 0.82–4.53, *p* = 0.132; non-significant), and HALP T1 (OR = 4.95, 95% CI 3.24–7.55, *p* < 0.001); GNRI < 98 showed a positive but non-significant association (OR = 3.22, 95% CI 0.77–13.47, *p* = 0.109), reflecting its near-universal at-risk classification. After adjustment for age, sex, and APACHE II score (Model 2), the association of mNUTRIC was substantially attenuated (OR = 2.16, 95% CI 1.02–4.61, *p* = 0.046). In the fully adjusted model (Model 3), only two indices retained independent associations with 28-day ICU mortality: HALP T1 (OR = 3.41, 95% CI 2.13–5.46, *p* < 0.001) and low PNI (OR = 2.48, 95% CI 1.09–5.66, *p* = 0.031). The association of mNUTRIC was fully attenuated after complete adjustment (OR = 1.73, 95% CI 0.79–3.80, *p* = 0.170), reflecting its inherent collinearity with the SOFA and APACHE II scores, which are direct components of its calculation. GNRI (OR = 2.18, 95% CI 0.49–9.73, *p* = 0.308) and INA (OR = 1.21, 95% CI 0.48–3.02, *p* = 0.684) did not reach significance in the fully adjusted model, a finding directly attributable to the ceiling effect whereby almost all patients—survivors and non-survivors alike—were classified as at-risk. Notably, the HALP association was the strongest and most robust across all three models. Full regression results are presented in Table 3.

**Table 3:** Multivariable logistic regression: association of nutrition-inflammation indices with 28-day ICU mortality.

Index	Model 1 OR (95%CI)	Model 1 *P*	Model 2 OR (95%CI)	Model 2 *P*	Model 3 OR (95%CI)	Model 3 *P*
mNUTRIC ≥5	5.95 (3.14–11.28)	< 0.001	2.16 (1.02–4.61)	0.046	1.73 (0.79–3.80)	0.170
PNI < 45	3.71 (1.69–8.13)	0.001	3.16 (1.41–7.09)	0.005	2.48 (1.09–5.66)	0.031
GNRI < 98	3.22 (0.77–13.47)	0.109	2.19 (0.51–9.48)	0.295	2.18 (0.49–9.73)	0.308
INA grade 3–4	1.93 (0.82–4.53)	0.132	1.30 (0.54–3.16)	0.556	1.21 (0.48–3.02)	0.684
HALP T1 (lowest tertile)	4.95 (3.24–7.55)	< 0.001	4.28 (2.76–6.65)	< 0.001	3.41 (2.13–5.46)	< 0.001

OR, odds ratio; CI, confidence interval. Model 1: unadjusted. Model 2: adjusted for age, sex, and APACHE II score. Model 3 (fully adjusted): additionally adjusted for SOFA score, acute kidney injury, Charlson Comorbidity Index, mechanical ventilation, and vasopressor use. mNUTRIC ≥5 = high nutritional risk; PNI < 45 = low prognostic nutritional index; GNRI < 98 = nutritional risk; INA grade 3–4 = combined hypoalbuminaemia and lymphopenia; HALP T1 = lowest tertile ( ≤ 171.11). HALP T1 and low PNI remained independently significant in the fully adjusted model.

### ROC analysis

To evaluate the discriminative performance of the five indices for 28-day ICU mortality, ROC analysis was performed in both cohorts (Figure 2, Table 4). In the derivation cohort, PNI achieved the highest discrimination (AUROC 0.761, 95% CI 0.712–0.808), followed closely by HALP (0.745, 95% CI 0.693–0.795) and mNUTRIC (0.723, 95% CI 0.673–0.769); GNRI (0.689) and INA (0.671) were the weakest discriminators. This ranking was reproduced in the external validation cohort, in which PNI (AUROC 0.742, 95% CI 0.657–0.824) and HALP (0.713, 95% CI 0.623–0.798) again performed best, with mNUTRIC (0.731) comparable and INA the weakest (0.619). The consistency of the index ranking across two independent cohorts supports PNI and HALP as the most discriminative single indices in critically ill patients with bone infections, and highlights the limited stand-alone discrimination of GNRI and INA in this population despite their strong univariate signal in less selected cohorts. The optimal cut-offs derived by Youden's index in the derivation cohort were mNUTRIC ≥ 6, PNI ≤ 35.15, GNRI ≤ 81.90, INA grade ≥ 4, and HALP ≤ 191.91. When applied to the external validation cohort, PNI and HALP maintained the most balanced sensitivity–specificity profiles (derivation: PNI sensitivity 0.694, specificity 0.712; HALP sensitivity 0.730, specificity 0.676; external validation: PNI sensitivity 0.756, specificity 0.688; HALP sensitivity 0.732, specificity 0.651; Table 2 and Supplementary Figure S6).

**Figure 2:**
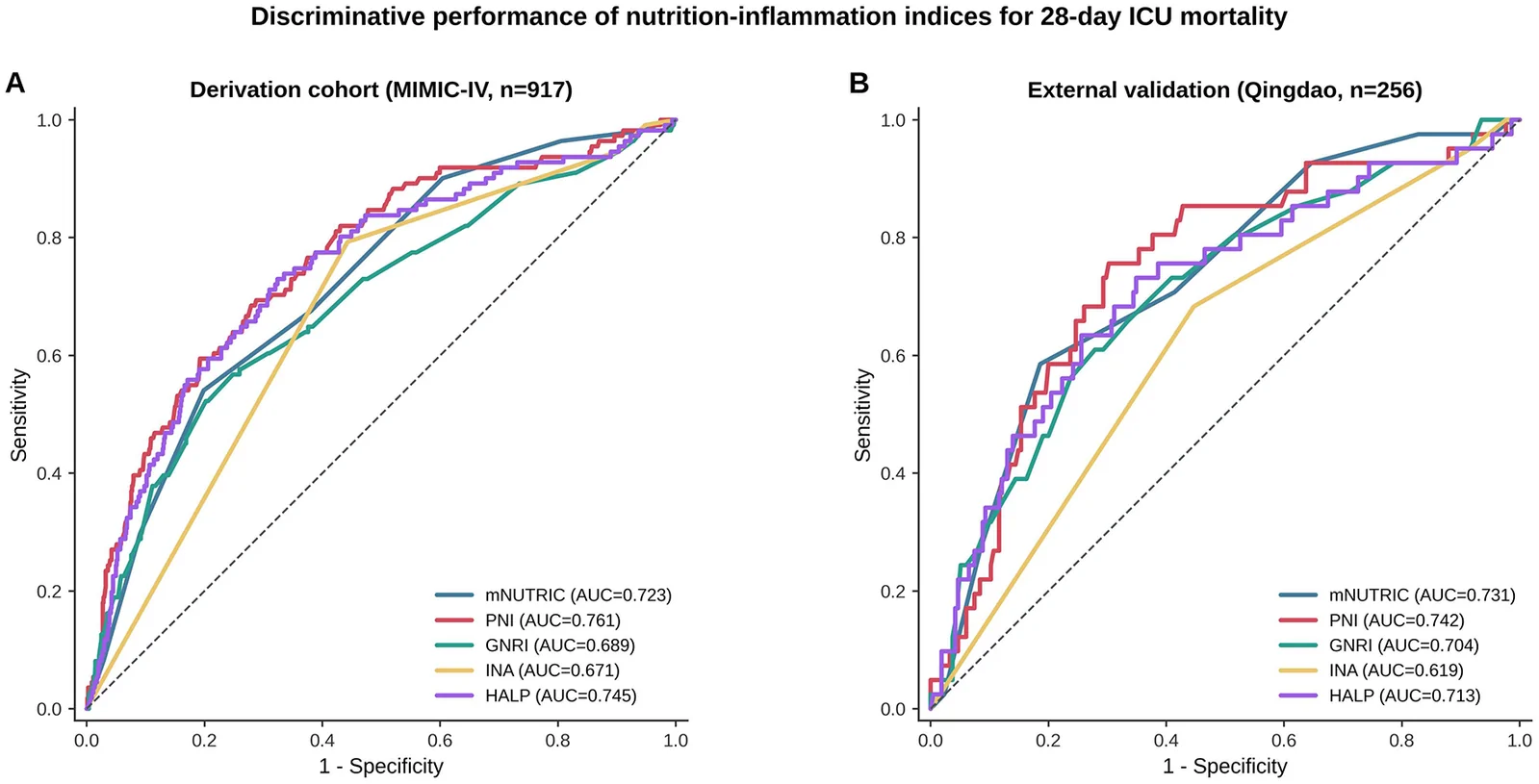
Receiver operating characteristic curves for five nutrition-inflammation indices predicting 28-day ICU mortality. **(A)** Derivation cohort (MIMIC-IV, *n* = 917); **(B)** external validation cohort (Affiliated Hospital of Qingdao University, *n* = 256). Each curve represents one index; area under the curve (AUC) values with 95% confidence intervals were estimated by 1,000 stratified bootstrap iterations. Protective indices were sign-inverted so that higher values denote higher risk. PNI and HALP achieved the highest discrimination in both cohorts.

**Table 4:** Discriminative performance of nutrition-inflammation indices (ROC analysis).

Index	Derivation cohort AUROC (95%CI)	External validation AUROC (95%CI)
mNUTRIC	0.723 (0.673–0.769)	0.731 (0.646–0.809)
PNI	0.761 (0.712–0.808)	0.742 (0.657–0.824)
GNRI	0.689 (0.632–0.742)	0.704 (0.608–0.790)
INA	0.671 (0.624–0.715)	0.619 (0.536–0.698)
HALP	0.745 (0.693–0.795)	0.713 (0.623–0.798)

AUROC, area under the receiver operating characteristic curve; 95% CI estimated by 1,000 stratified bootstrap iterations. Derivation cohort: MIMIC-IV bone infections ICU patients (*n* = 917; 28-day mortality 12.1%). External validation cohort: affiliated hospital of Qingdao university (*n* = 256; 28-day mortality 16.0%). Protective indices were sign-inverted so that higher values denote higher risk. PNI achieved the highest AUROC in both cohorts, followed by HALP. mNUTRIC cutoff ≥5 (derivation) and ≥4 (external).

### Restricted cubic spline analysis

To examine whether nonlinear dose-response relationships exist between the continuous indices and 28-day ICU mortality, RCS analysis was performed (Figure 3). All four continuous indices (mNUTRIC, PNI, GNRI, and HALP) showed a significant overall association with mortality (all *p*-overall < 0.001). The HALP score exhibited a strong and statistically significant nonlinear relationship (*p*-nonlinearity < 0.001): mortality risk rose steeply as HALP decreased below approximately 150–200 and then plateaued at higher values, indicating a clinically actionable low-HALP threshold. In contrast, mNUTRIC (*p*-nonlinearity = 0.802), PNI (*p*-nonlinearity = 0.105), and GNRI (*p*-nonlinearity = 0.117) displayed predominantly monotonic, approximately linear relationships with risk over their observed ranges. The pronounced threshold behavior of HALP is consistent with its independent and robust association in the multivariable models and provides a biologically plausible basis for tertile-based risk stratification.

**Figure 3:**
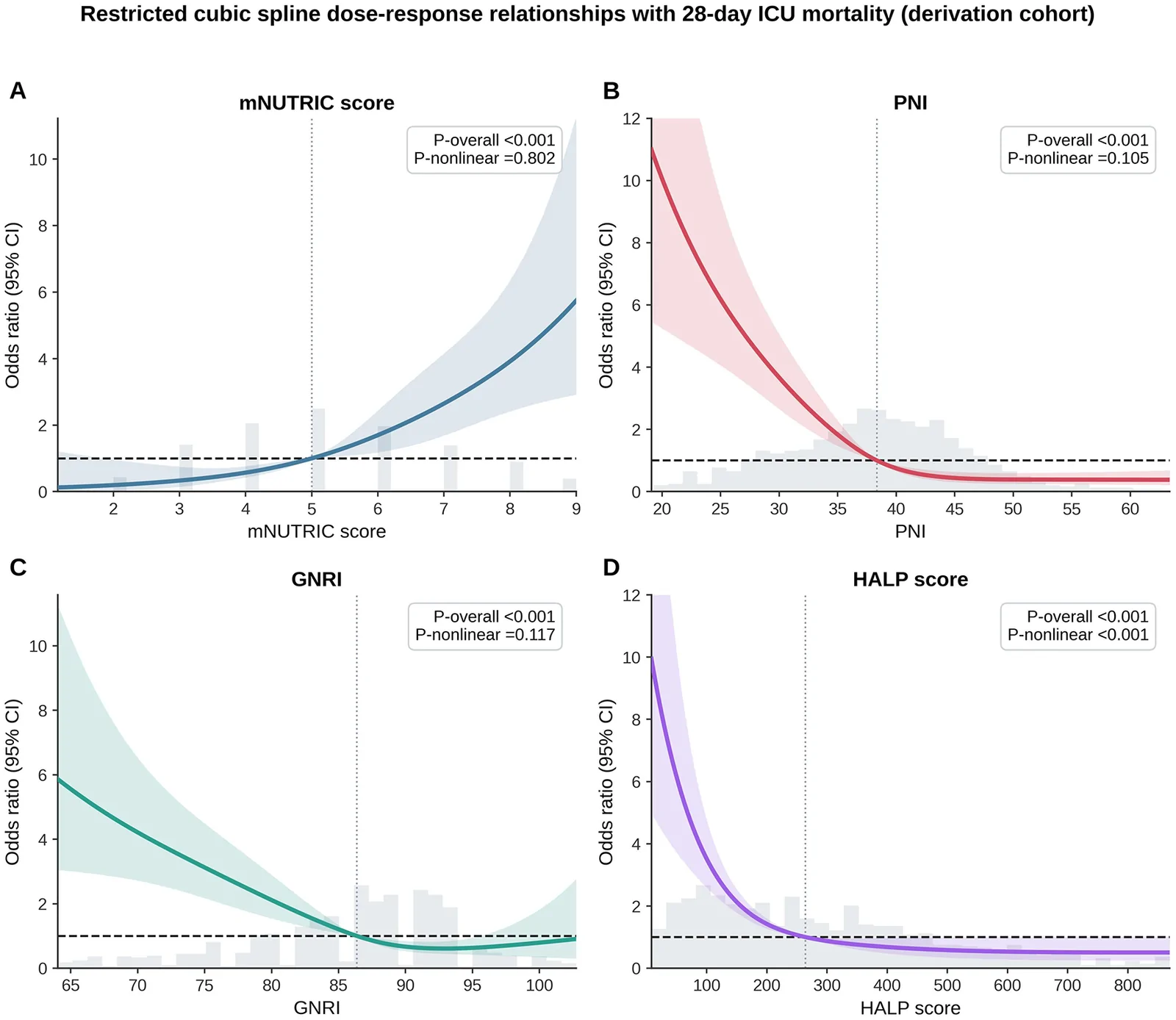
Restricted cubic spline analysis of the association between four continuous nutrition-inflammation indices and 28-day ICU mortality. Note: Each panel displays the odds ratio (OR) with its 95% confidence interval (shaded ribbon) as a function of the continuous index value, relative to the cohort median (reference OR=1, dashed horizontal line). Four knots were placed at the 5th, 35th, 65th, and 95th percentiles of each distribution. The underlying distribution of each index is shown as a histogram at the bottom of each panel; the x-axis is clipped to the 1st−−99th percentile for clarity. **(A)** mNUTRIC; **(B)** PNI; **(C)** GNRI; **(D)** HALP. The HALP score showed a significant nonlinear threshold relationship (*p*–nonlinearity < 0.001), whereas mNUTRIC, PNI, and GNRI were approximately linear.

### Subgroup analysis

Given its robust independent association and clear nonlinear threshold, the HALP T1–mortality relationship was further examined across clinical subgroups (Figure 4, Table 5). The association of HALP T1 with 28-day ICU mortality was remarkably consistent across all strata, with ORs of approximately 4 and statistical significance retained in every subgroup defined by age (< 70 vs. ≥70 years), sex, diabetes mellitus, chronic kidney disease, and heart failure, and no evidence of effect modification (all interaction *p* > 0.27). The only significant interaction was observed for AKI (interaction *p* = 0.002): the HALP effect was very large among patients without AKI (OR = 13.68) and attenuated but still significant among patients with AKI (OR = 2.23, *p* = 0.002), most plausibly because AKI itself is a dominant driver of mortality that partially subsumes the nutritional signal. In contrast, the subgroup estimates for PNI < 45 were unstable due to sparse data, and no claim of cross-subgroup consistency is made for PNI. Overall, the consistency of the HALP association across heterogeneous clinical subgroups reinforces its value as a generalizable prognostic marker in this population.

**Figure 4:**
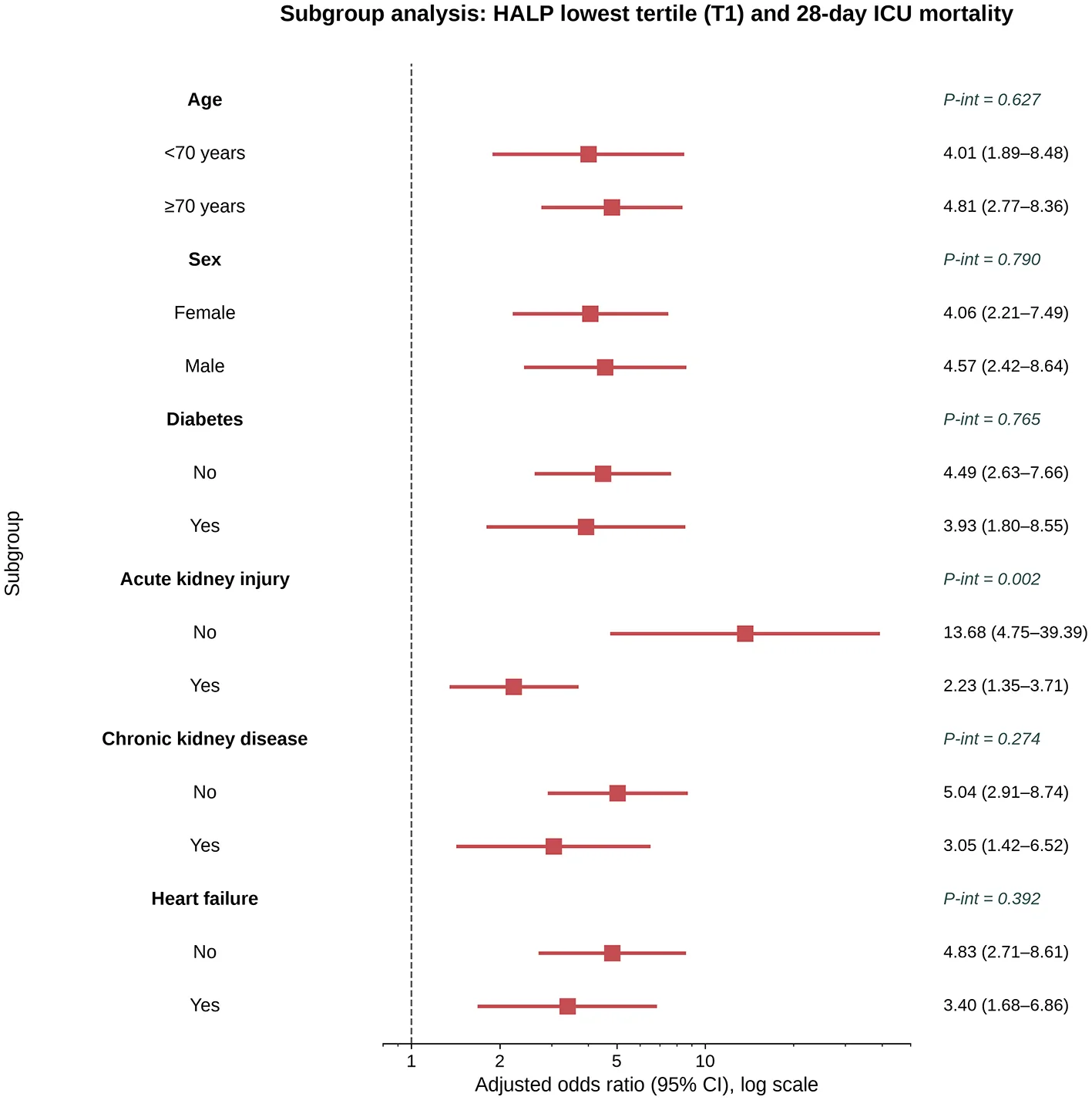
Subgroup analysis of the association between HALP lowest tertile (T1) and 28-day ICU mortality. Each square represents the OR for the corresponding subgroup, with horizontal lines indicating the 95% confidence interval. All models were adjusted for age, sex, and APACHE II score, excluding the stratifying variable. The *p*-value for interaction tests for effect modification. The HALP T1 association was consistent across all subgroups (all interaction *p* > 0.27) except acute kidney injury.

**Table 5:** Subgroup analysis: association of HALP T1 and PNI < 45 with 28-day ICU mortality.

Exposure/subgroup	*N* (events)	OR (95%CI)	*P*-value	*P*-int
HALP T1 (lowest tertile)				
Age				0.627
< 70 years	400 (37)	4.01 (1.89–8.48)	< 0.001	
>=70 years	517 (74)	4.81 (2.77–8.36)	< 0.001	
Sex				0.790
Female	405 (58)	4.06 (2.21–7.49)	< 0.001	
Male	512 (53)	4.57 (2.42–8.64)	< 0.001	
Diabetes				0.765
No	599 (76)	4.49 (2.63–7.66)	< 0.001	
Yes	318 (35)	3.93 (1.80–8.55)	< 0.001	
Acute kidney injury				0.002
No	521 (21)	13.68 (4.75–39.39)	< 0.001	
Yes	396 (90)	2.23 (1.35–3.71)	0.002	
Chronic kidney disease				0.274
No	712 (68)	5.04 (2.91–8.74)	< 0.001	
Yes	205 (43)	3.05 (1.42–6.52)	0.004	
Heart failure				0.392
No	636 (59)	4.83 (2.71–8.61)	< 0.001	
Yes	281 (52)	3.40 (1.68–6.86)	< 0.001	
**PNI**<**45**				
Age				0.166
< 70 years	400 (37)	1.62 (0.53–4.91)	0.398	
>=70 years	517 (74)	5.31 (1.59–17.73)	0.007	
Sex				0.445
Female	405 (58)	2.29 (0.78–6.78)	0.133	
Male	512 (53)	4.37 (1.28–14.90)	0.018	
Diabetes				0.182
No	599 (76)	2.23 (0.92–5.42)	0.077	
Yes	318 (35)	9.12 (1.17–71.23)	0.035	
Acute kidney injury				0.030
No	521 (21)	–	–	
Yes	396 (90)	1.83 (0.77–4.32)	0.169	
Chronic kidney disease				0.487
No	712 (68)	2.62 (1.08–6.40)	0.034	
Yes	205 (43)	5.52 (0.71–43.22)	0.104	
Heart failure				0.812
No	636 (59)	3.27 (1.14–9.38)	0.027	
Yes	281 (52)	2.89 (0.81–10.25)	0.101	

OR, odds ratio; CI, confidence interval; *P*-int, *p*-value for interaction. All models were adjusted for age, sex, and APACHE II score, excluding the stratifying variable. *N* (events) denotes the number of patients (and 28-day deaths) in each subgroup. – = model not estimable owing to zero events in a cell. The HALP T1 association was consistent across all subgroups (all interaction *p* > 0.27) except acute kidney injury. In contrast, the PNI < 45 subgroup estimates were unstable due to sparse data (zero-event cell in the no-AKI stratum), and no claim of cross-subgroup consistency is made for PNI.

### Machine learning model development and performance evaluation

To develop a predictive model for 28-day ICU mortality, a dual LASSO-Boruta feature selection strategy was applied to the 71 candidate features shared between the two cohorts (Supplementary Figures S1, S2, Supplementary Table S2). LASSO retained 24 features at the optimal regularization parameter, and Boruta confirmed 28 features; their intersection yielded 14 final features (EPV = 7.9): age, AKI, APS III, Charlson Comorbidity Index, albumin, chloride, fibrinogen, glucose, lymphocyte percentage, PO_2_, RDW, red blood cell count, SpO_2_, and mNUTRIC high-risk status. Notably, mNUTRIC was the only composite nutrition-inflammation index selected into the final model, whereas the individual laboratory constituents of the other indices (albumin, lymphocytes, hemoglobin-related RDW and red blood cell count) were retained in their own right. Five ML models were trained with SMOTE and five-fold stratified cross-validation and evaluated externally (Figure 5, Table 6). All models discriminated well; on external validation, LightGBM achieved the highest AUROC (0.956, 95% CI 0.921–0.985), followed closely by XGBoost (0.954) and RF (0.937), with LR (0.885) and SVM (0.923) performing slightly lower. The gradient-boosting models also achieved the best external calibration (Brier score 0.045–0.046) and precision-recall performance (AUPRC 0.83). The external AUROC exceeded the cross-validation AUROC for the tree-ensemble models; this gap was investigated and attributed to the greater intrinsic separability of the external cohort rather than to information leakage, as per-feature univariate AUROCs were near-identical across the two cohorts. Under the leakage-free nested cross-validation design in which feature selection was repeated independently within each training fold, the nested cross-validation AUROC was 0.829 (fold range 0.803–0.849), and the five resulting fold-models achieved external-validation AUROCs of 0.914–0.960 (Supplementary Table S8), confirming that the external performance of the model is not inflated by the feature-selection procedure. Model calibration curves are shown in Supplementary Figure S3.

**Figure 5:**
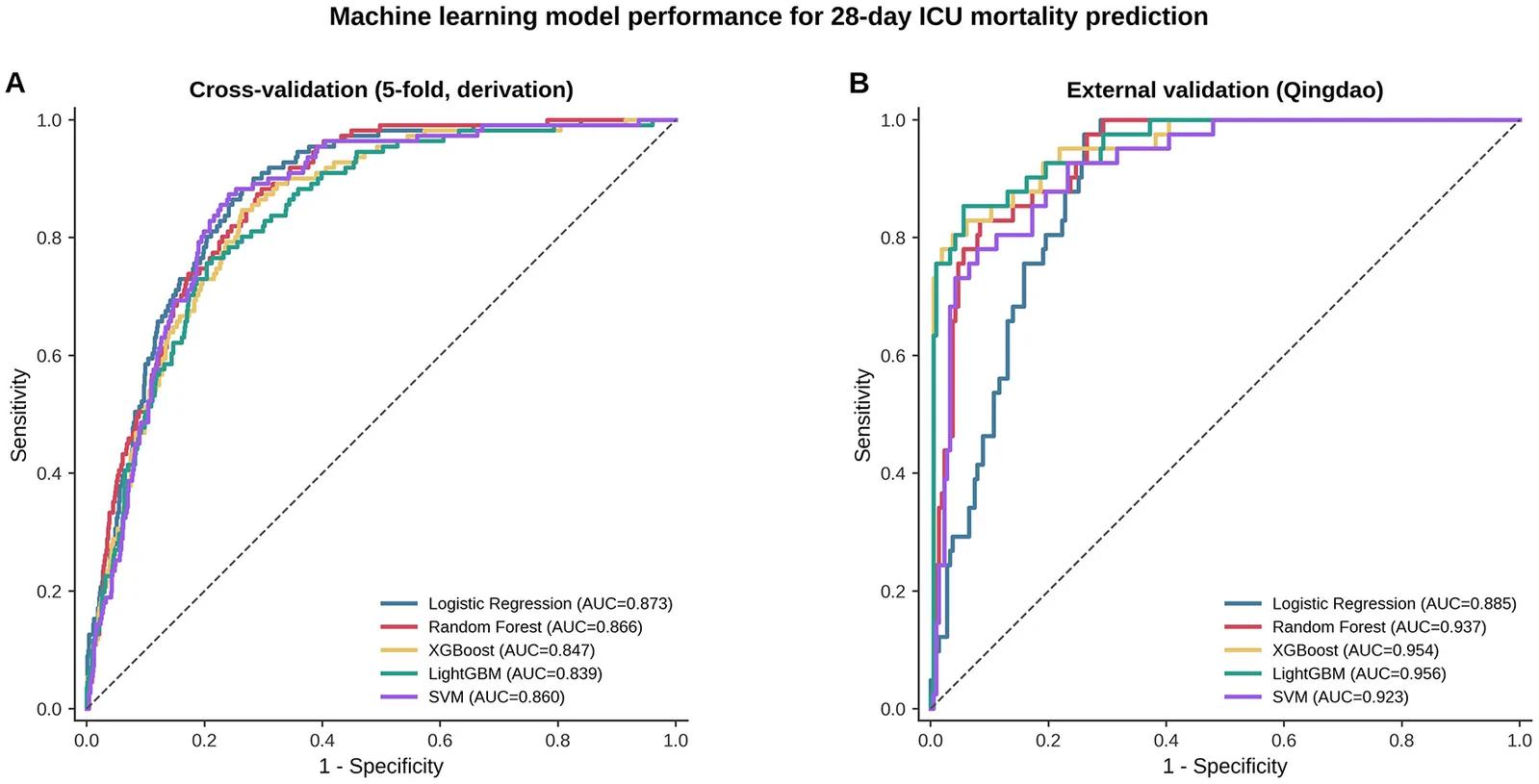
Receiver operating characteristic curves for five machine learning models predicting 28-day ICU mortality. **(A)** Cross-validation performance (five-fold stratified). **(B)** External validation performance. Shaded bands indicate 95% confidence intervals for the external AUROC (1,000 bootstrap iterations). LightGBM achieved the highest external AUROC (0.956). LR, logistic regression; RF, random forest; SVM, support vector machine.

**Table 6:** Machine learning model performance: derivation (5-fold CV) and external validation.

Model	CV AUROC	CV AUPRC	CV brier	External AUROC (95%CI)	External AUPRC	External brier
Logistic regression	0.875	0.488	0.140	0.885 (0.840–0.923)	0.522	0.140
Random forest	0.867	0.443	0.109	0.937 (0.902–0.967)	0.709	0.077
XGBoost	0.848	0.436	0.135	0.954 (0.917–0.984)	0.831	0.046
LightGBM	0.841	0.433	0.115	0.956 (0.921–0.985)	0.830	0.045
SVM	0.860	0.438	0.108	0.923 (0.879–0.961)	0.656	0.086

CV, five-fold stratified cross-validation with SMOTE (synthetic minority over-sampling technique) applied within each training fold to address class imbalance (event rate 12.1%). AUROC, area under the receiver operating characteristic curve; AUPRC, area under the precision-recall curve; Brier score = mean squared prediction error (lower is better). 95% CI for external AUROC estimated by 1,000 bootstrap iterations. LightGBM achieved the highest external AUROC (0.956). EPV = 7.9 (111 events/14 final features). All models were trained on the MIMIC-IV derivation cohort (*n* = 917) and validated on the independent external cohort (*n* = 256).

### SHAP analysis

To elucidate the predictive mechanisms of the model, SHAP interpretability analysis was performed directly on the final LightGBM model (Figure 6; Supplementary Table S3). The most influential predictors in the derivation cohort, ranked by mean absolute SHAP value, were APS III score (higher → higher risk), acute kidney injury (present → higher risk), arterial oxygen tension (PO_2_; higher → lower risk), peripheral oxygen saturation (SpO_2_; higher → lower risk), and the Charlson Comorbidity Index (higher → higher risk). Lymphocyte percentage (higher → lower risk), RDW (higher → higher risk), age (higher → higher risk), and albumin (higher → lower risk) also ranked among the leading features. The ranking was highly consistent with the previously reported random-forest analysis (Spearman ρ = 0.587) and was almost perfectly preserved in the external validation cohort (ρ = 0.987, *p* < 0.001), confirming that the model's prediction mechanism is stable across cohorts. Importantly, three of the leading predictors (lymphocyte percentage, albumin, and RDW) are direct constituents of the nutrition-inflammation indices, providing a mechanistic bridge between the index-based and ML analyses: the same nutritional-immune axis that underlies the prognostic value of HALP and PNI also drives the predictions of the best-performing ML model. The composite mNUTRIC feature contributed modestly (rank 11), consistent with its collinearity with the severity scores already represented by APS III.

**Figure 6:**
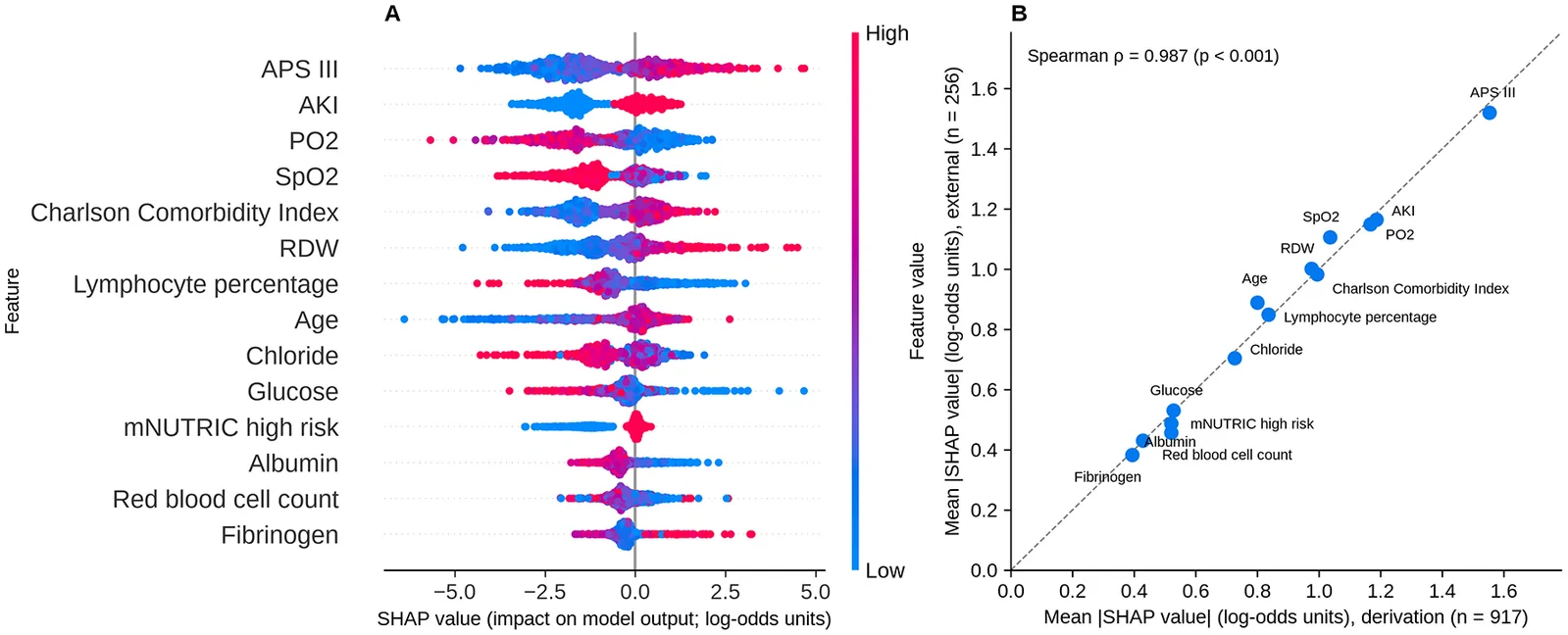
SHAP analysis of the final LightGBM model. **(A)** Beeswarm plot of SHAP values in the derivation cohort (*n* = 917); each point is one patient, colored by the feature value (blue = low, red = high). **(B)** Stability of feature importance between the derivation and external validation (*n* = 256) cohorts; each point is one of the 14 model features, and the dashed line denotes identity.

### Calibration and decision curve analysis

To evaluate calibration and clinical utility, calibration-curve analysis and DCA were performed (Supplementary Figures S4, S5). The gradient-boosting models were well calibrated on external validation, with low Brier scores (LightGBM 0.045; XGBoost 0.046). Decision curve analysis demonstrated that the best ML model provided greater net clinical benefit than any single nutrition-inflammation index across the clinically relevant range of threshold probabilities; at a 10% threshold, the net benefit of the ML model (0.129) exceeded that of PNI (0.093) and HALP (0.075). These findings indicate that, although PNI and HALP are the most useful single-marker tools, an integrated ML model incorporating these nutritional constituents alongside severity and physiological variables offers superior discrimination and clinical utility for early risk stratification.

### Multicollinearity diagnostics and component-aware sensitivity analyses

VIF values for all covariates in the fully adjusted model ranged from 1.01 to 1.90, with the highest values observed for APACHE II (1.90) and SOFA (1.82), all well below the prespecified threshold of 5, indicating no problematic multicollinearity. The Box–Tidwell test for the linearity-in-the-logit assumption yielded non-significant interaction terms for all continuous covariates (all *p* > 0.2), confirming that the assumption was not violated. In component-aware sensitivity models, the association of the HALP lowest tertile with 28-day ICU mortality remained robust across all specifications (OR 3.32–3.94, all *p* < 0.001), as did the association of low PNI (OR 2.42–2.74, all *p* ≤ 0.034). For mNUTRIC, a component-aware model adjusting only for non-embedded covariates (sex, acute kidney injury, mechanical ventilation, and vasopressor use) revealed a significant association with mortality (OR = 3.93, 95% CI 1.99–7.78, *p* < 0.001), indicating that the attenuation observed in the original fully adjusted model reflected over-adjustment for the score's own embedded components rather than a true absence of association. GNRI and INA remained non-significant across all sensitivity specifications. Detailed results are provided in Supplementary Tables S5–S7.

## Discussion

The present study provides the first systematic head-to-head comparison of five nutrition-inflammation indices for predicting 28-day ICU mortality in critically ill patients with bone infections, using a MIMIC-IV derivation cohort (*n* = 917) with external validation in an independent cohort from the Affiliated Hospital of Qingdao University (*n* = 256). The principal findings were as follows: (1) after full adjustment for disease severity and comorbidity, HALP T1 and low PNI remained independently associated with 28-day ICU mortality, whereas mNUTRIC, GNRI, and INA did not; (2) HALP displayed a strong, statistically significant nonlinear threshold relationship with mortality; (3) PNI and HALP exhibited the strongest and most consistent discriminative performance in both cohorts (PNI AUROC 0.761/0.742; HALP 0.745/0.713); (4) the HALP association was consistent across all clinical subgroups; and (5) an interpretable ML model integrating 14 routinely available features achieved excellent external discrimination (LightGBM AUROC 0.956), with PO_2_, APS III, and lymphocyte percentage identified as the principal predictors by SHAP analysis, and superior net clinical benefit over any single index by DCA.

The robust, independent, and strongly nonlinear association of the HALP score is the central finding of this study. HALP integrates four inexpensive and universally available parameters—hemoglobin, albumin, lymphocyte count, and platelet count—that jointly capture anemia of inflammation, hypoalbuminaemia, immune depletion, and the platelet-mediated acute-phase response (28–30). In critically ill patients with bone infections, in whom chronic infection and protein-energy wasting are tightly coupled (10, 13, 14), this multi-domain construct appears particularly well suited to capture host vulnerability. Our observation of a low-HALP threshold, below which mortality risk rises steeply, is consistent with prior reports in cancer and, more recently, in sepsis, where low HALP independently predicted 28-day mortality in a MIMIC-IV cohort (32). The consistency of the HALP effect across age, sex, diabetes, chronic kidney disease, and heart failure subgroups further supports its generalizability as a prognostic marker in this population.

The independent association of low PNI, and its strong and consistent discrimination in both cohorts, aligns with a substantial literature demonstrating the prognostic value of PNI in sepsis, ventilator-associated pneumonia, and diabetic foot infection (21–23). PNI and HALP share albumin and lymphocyte terms, and their strong inter-correlation explains why both, but not necessarily all of their individual components, remained informative. That both albumin- and lymphocyte-based indices outperformed the weight-based GNRI underscores the primacy of the inflammatory-immune axis over anthropometric nutritional reserve in the acute critical-illness setting of bone infections.

A key and clinically instructive contrast with prior work concerns GNRI. In cardiothoracic surgery ICU populations, GNRI has been reported as the most consistent independent predictor of 28-day mortality, with a clinically actionable nonlinear threshold. In the present bone infections cohort, by contrast, GNRI lost both independent association and stand-alone discrimination. The explanation is a pronounced ceiling effect: 94.9% of the derivation cohort—and 98.2% of non-survivors vs. 94.4% of survivors—were classified as at-risk by GNRI (< 98), leaving little dynamic range to separate outcomes. A similar, though less extreme, ceiling affected INA (90.6% high-risk). This finding is a cautionary illustration that the discriminative performance of a nutrition-inflammation index is population-dependent: an index optimized in one case-mix may be uninformative in another if the underlying risk factor is near-universal. In bone infections, where hypoalbuminaemia is almost ubiquitous, indices that preserve continuous resolution (HALP, PNI) outperform dichotomized, albumin-and-weight-dominated indices (GNRI, INA).

The attenuation of mNUTRIC after full adjustment (OR = 1.73, *p* = 0.170) is methodologically expected and mirrors observations in other ICU cohorts (18, 20). Because the SOFA and APACHE II scores are direct components of the mNUTRIC calculation, adjusting for these severity measures removes much of the score's predictive information; the residual nutritional signal in mNUTRIC is comparatively small. Consistent with this, mNUTRIC was the only composite index selected by both LASSO and Boruta into the ML model, yet contributed the least among the 14 features in SHAP analysis—its information is largely shared with the severity scores that the model already contains.

The interpretable ML model achieved excellent external discrimination (LightGBM AUROC 0.956), exceeding the performance of any single index and comparable to or better than recent ML mortality models in critically ill populations (34–36). The higher external than cross-validation AUROC observed for the tree-ensemble models could superficially suggest optimism or leakage; however, we ruled this out by confirming that per-feature univariate AUROCs were near-identical across the two cohorts, indicating that the external cohort was simply intrinsically more separable (for example, a stronger albumin and lymphocyte signal). Reassuringly, the SHAP analysis grounds the model in physiologically coherent predictors: oxygenation (PO_2_, SpO_2_), acute physiological derangement (APS III), the nutritional-immune axis (lymphocyte percentage, albumin), and hematological stress (RDW). The convergence of the index-based and ML analyses on the same albumin-lymphocyte biology strengthens the mechanistic interpretation and the case for incorporating these inexpensive markers into early risk-stratification tools.

The clinical implications are threefold. First, HALP and PNI, both computable at no incremental cost from an admission complete blood count and albumin, can be used for rapid bedside risk stratification in critically ill patients with bone infections, with the low HALP tertile identifying a group at roughly three- to five-fold higher risk of 28-day mortality. Second, our results caution against the uncritical transfer of a nutrition-inflammation index across populations. Third, an interpretable ML model integrating these nutritional constituents with severity and physiological variables offers superior discrimination and net clinical benefit and, if prospectively validated, could function as a decision-support tool for early identification of high-risk patients who may benefit from intensified nutritional, metabolic, and infection-directed care.

Several limitations should be acknowledged. First, the retrospective design precludes causal inference, and unmeasured confounders may have influenced the observed associations. Second, the number of events was modest (111 in the derivation cohort, 41 in the external validation cohort), yielding an events-per-variable ratio of 7.9 for the ML model in the derivation cohort. Although the 1-in-10 EPV heuristic is a traditional rule of thumb for model development—and, as van Smeden et al. have argued, lacks a universal rationale and depends on multiple factors including the strength of associations and outcome prevalence—it does not directly apply to external validation, where no parameters are estimated and the relevant consideration is the precision of performance estimates. The external estimate was imprecise as expected for 41 events (95% CI 0.921–0.985), and the fully nested cross-validation (with feature selection repeated within each training fold) reproduced external AUROCs of 0.914–0.960, arguing against leakage as the explanation for the high performance. Nevertheless, the EPV is below the conventional target of 10, and confirmation in larger, prospectively collected cohorts is required before clinical deployment (37–39). Fourth, the external validation cohort employed a four-component mNUTRIC (excluding the days-to-ICU component), which is not fully comparable to the five-component derivation score; although a harmonized sensitivity analysis showed that this definition shift did not materially affect the results—with near-identical classification rates, AUROCs, and fully adjusted associations—the cross-cohort comparison of mNUTRIC should be interpreted with this methodological difference in mind. Fifth, fibrinogen was missing in approximately 29% of derivation-cohort patients and was median-imputed, which may have diluted its contribution. Sixth, both cohorts identified bone infections by diagnosis codes without central adjudication of anatomical site, chronicity, or microbiology, limiting subgroup resolution. Finally, although the ML model achieved excellent external discrimination, its clinical implementation requires prospective validation within a real-time decision-support framework, including assessment of workflow integration and impact on patient outcomes.

## Conclusion

Among five nutrition-inflammation indices evaluated in critically ill patients with bone infections, HALP and PNI were the leading independent predictors of 28-day ICU mortality. HALP showed a robust, strongly nonlinear threshold effect that was consistent across all clinical subgroups and adjustment models, whereas GNRI—dominant in cardiothoracic populations—lost discriminative value here owing to a near-universal at-risk classification. An interpretable machine learning model integrating 14 routinely available clinical features achieved excellent external discrimination (AUROC = 0.956) and superior clinical utility, and SHAP analysis confirmed that the nutritional-immune axis central to HALP and PNI also drives the model's predictions. These findings support the use of HALP and PNI for rapid bedside risk stratification and, pending prospective validation, the deployment of an interpretable ML model as a decision-support tool for early identification of high-risk patients with bone infections in the ICU.

## Data Availability

The datasets presented in this study can be found in online repositories. The names of the repository/repositories and accession number(s) can be found in the article/Supplementary material.
