# Supplementary material for: Comparative evaluation of five nutrition-inflammation indices for predicting 28-day ICU mortality in critically ill patients with bone infections: a dual-cohort study with external validation and interpretable machine learning
Source: Front Nutr. 2026 Sep 16;13:1949102. doi: 10.3389/fnut.2026.1949102 (PMC13623587; doi:10.3389/fnut.2026.1949102)
Supplement: Supplementary file 1 [file Supplementary_File_1.pdf]

Supplementary Figure Legends

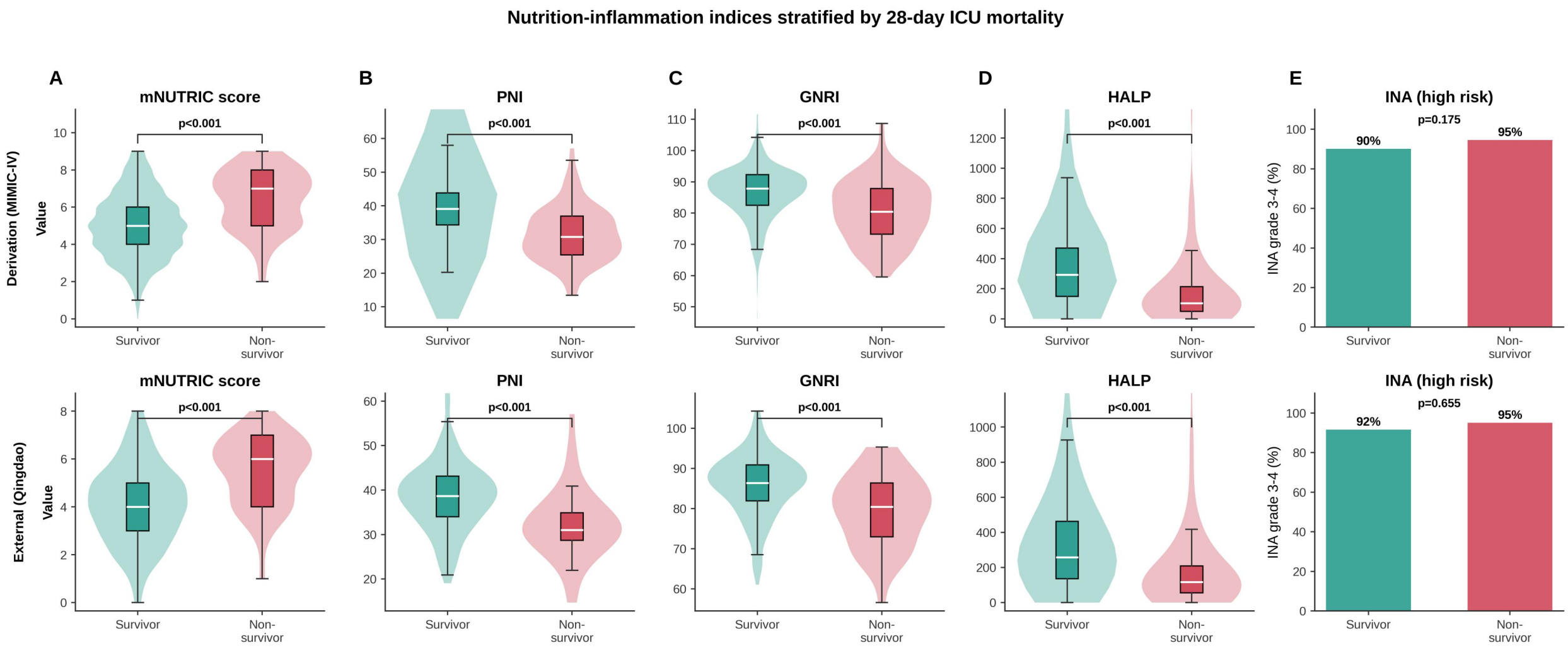

Supplementary Figure S1: Distribution of five nutrition-inflammation indices stratified by 28-day ICU survival status in the derivation cohort.

Note: *Violin plots with embedded box plots are shown for the continuous indices (mNUTRIC, PNI, GNRI, HALP); bar charts show the proportions for the categorical classifications. Non-survivors had systematically lower PNI, GNRI, and HALP and higher mNUTRIC than survivors (all p<0.001).*

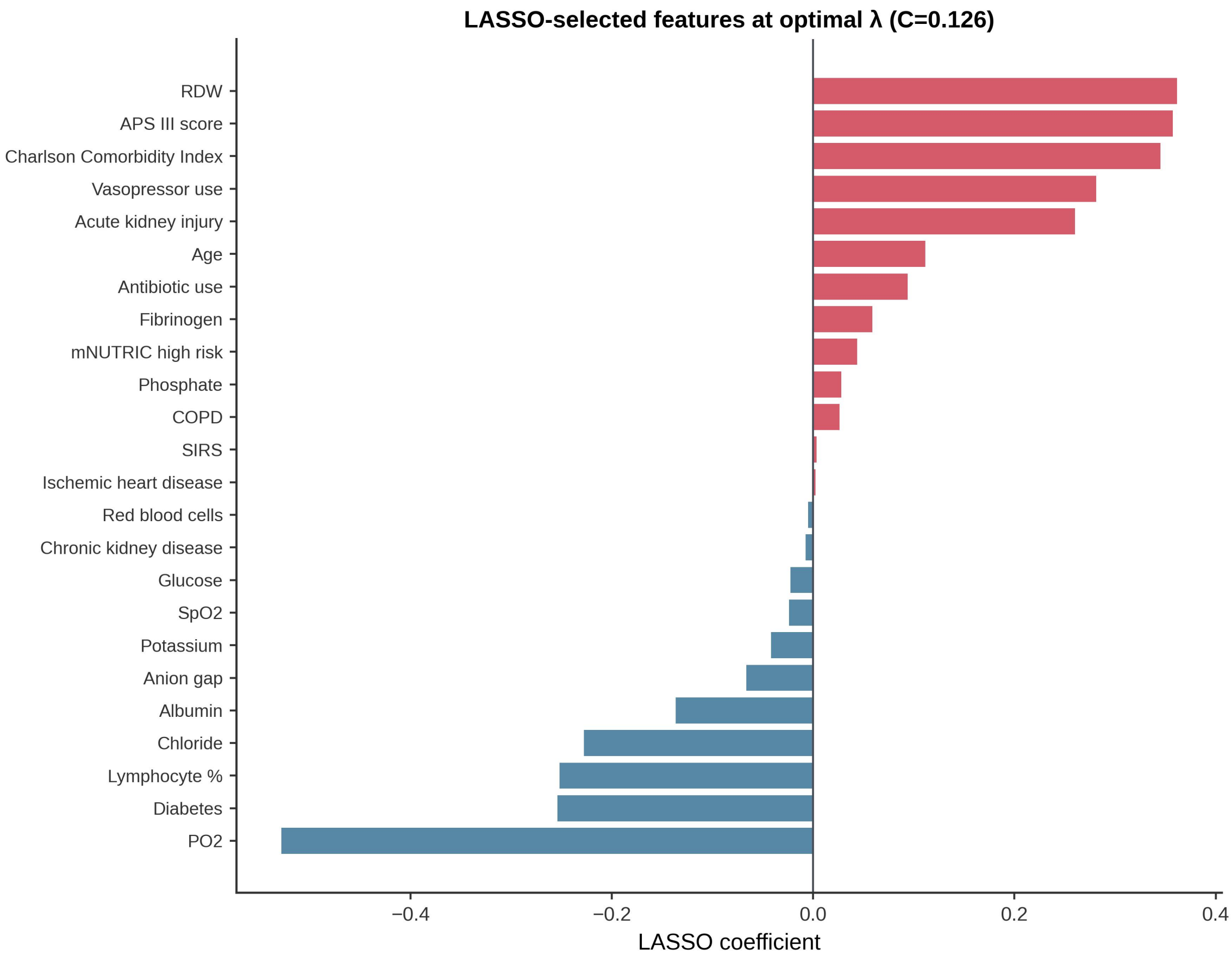

Supplementary Figure S2: LASSO regularization path and selected features.

Note: *Coefficients of the features retained at the optimal regularization parameter (λ) selected by cross-validation in the derivation cohort.*

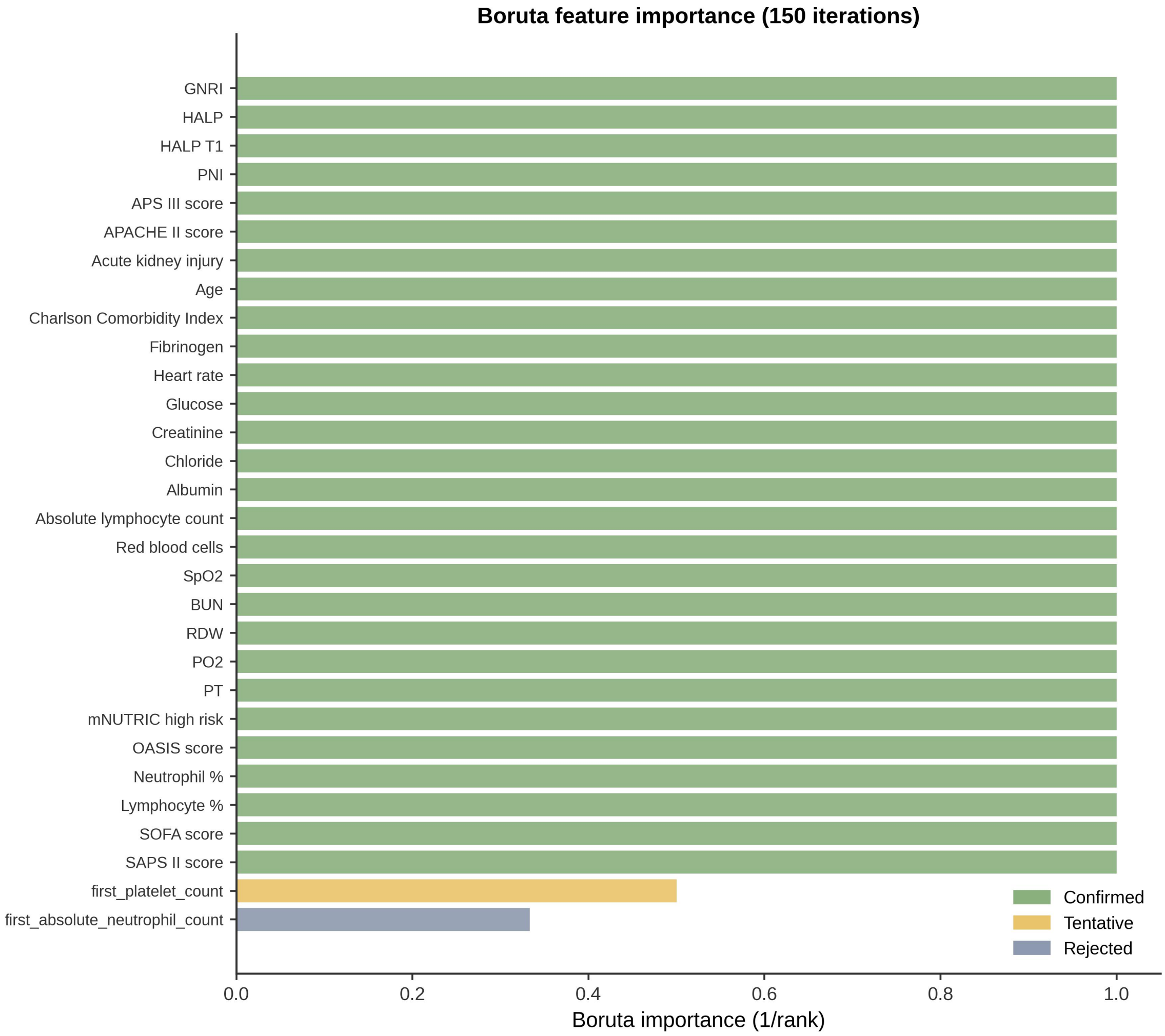

Supplementary Figure S3: Boruta feature importance analysis.

Note: *Candidate features ranked by the Boruta algorithm (150 iterations), which iteratively compares each real feature against randomized shadow features. The x-axis shows the importance expressed as the reciprocal of the final Boruta rank (1/rank; longer bars = higher importance). Bars are colored by the Boruta decision: confirmed (retained), tentative, or rejected. Only confirmed features were carried forward, and the final predictor set was defined as the intersection of the Boruta-confirmed and LASSO-selected features.*

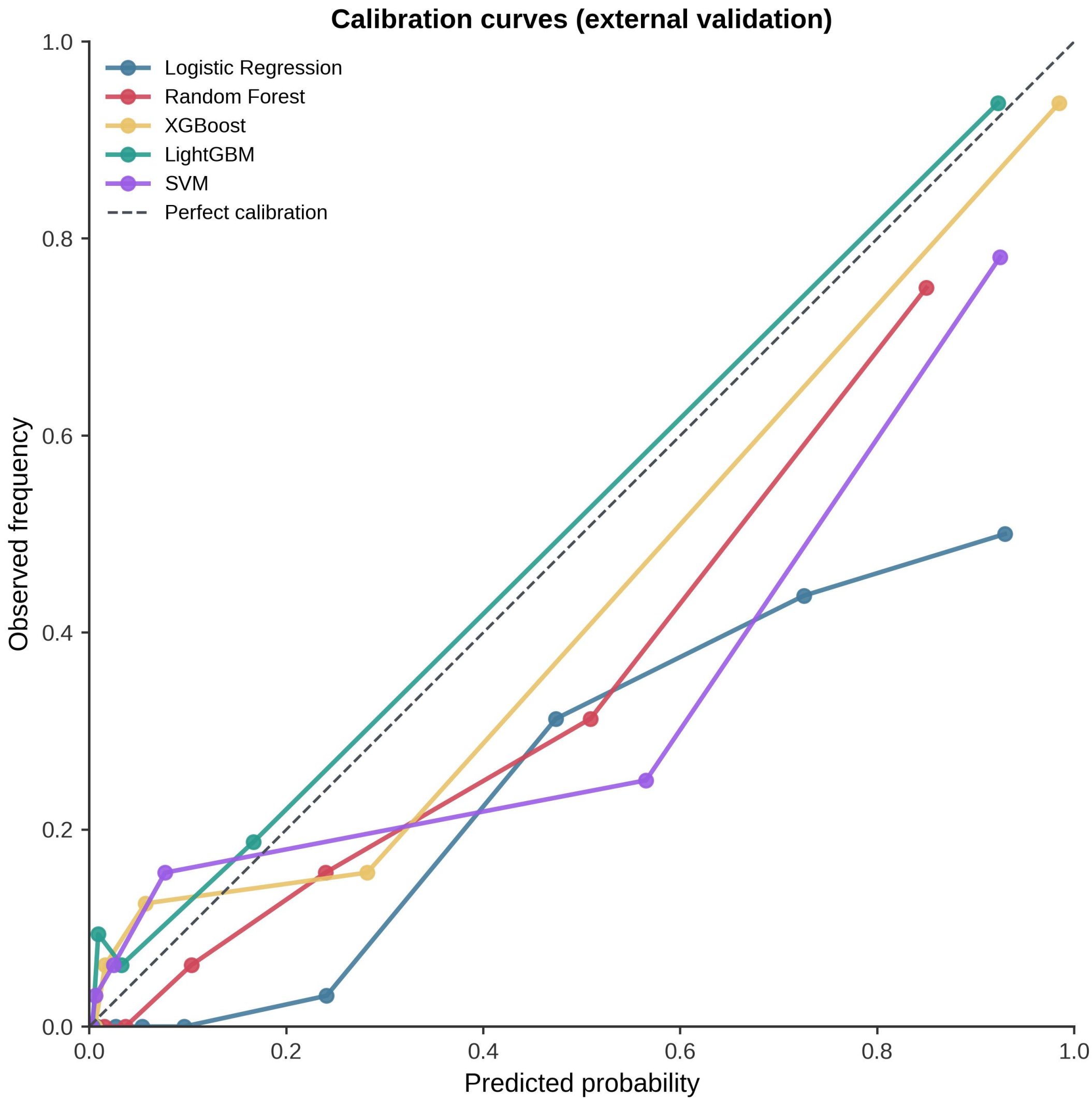

Supplementary Figure S4: Calibration curves for five machine learning models on external validation.

Note: *Each curve represents the relationship between predicted probability and observed event rate for one model. The diagonal represents perfect calibration. The gradient-boosting models (XGBoost, LightGBM) were best calibrated (Brier score 0.045–0.046).*

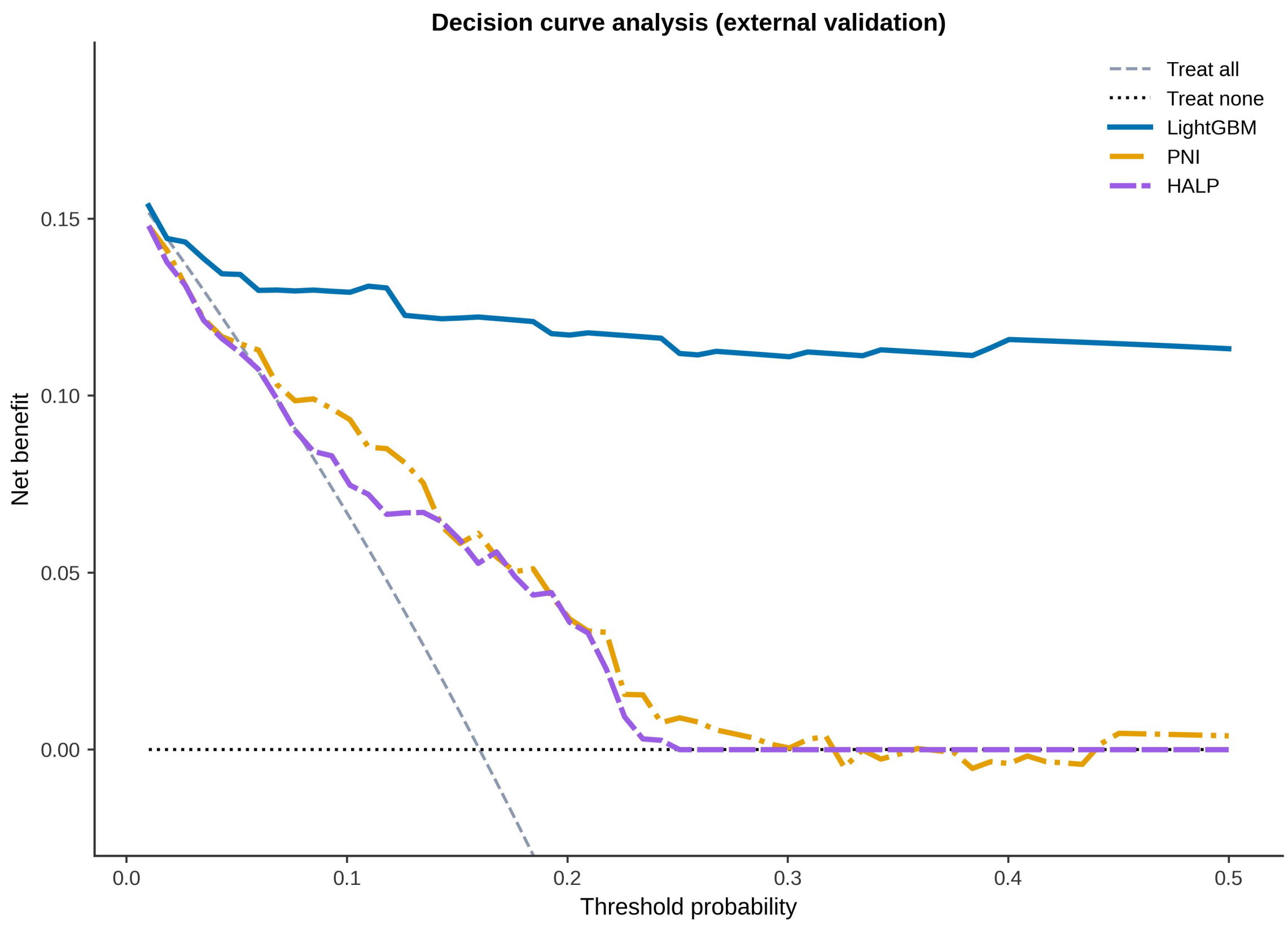

Supplementary Figure S5: Decision curve analysis for nutrition-inflammation indices and the optimal machine learning model.

Note: *The y-axis represents net benefit; the x-axis represents threshold probability. The "Treat All" line assumes all patients are treated and the "Treat None" line assumes none are treated. The machine learning model provided greater net benefit than any single index across the clinically relevant threshold range.*

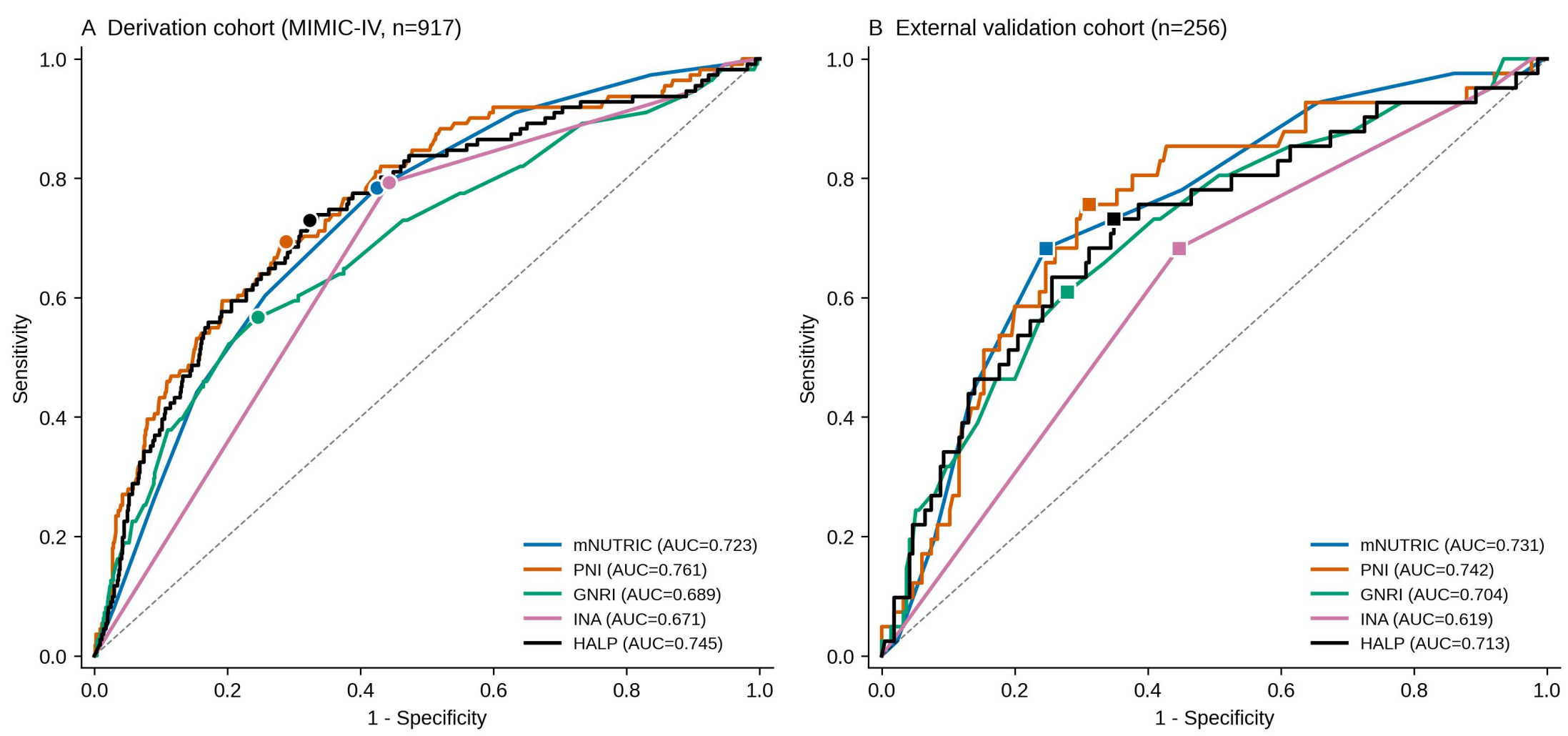

Figure S6: Receiver operating characteristic curves of the five nutrition–inflammation indices for 28-day ICU mortality with optimal cut-off points marked.

Note: (A) Derivation cohort (MIMIC-IV, n = 917); circles mark the optimal cut-off points determined by Youden's index. (B) External validation cohort (n = 256); squares mark the performance at the derivation-cohort cut-offs. AUROC values in the legends are as reported in Table 3 of the manuscript.

Supplementary Tables

Table S1: External Validation Cohort Baseline Characteristics

Variable	Non-survivors (n=41)	Survivors (n=215)	P-value
**Demographics**			
Age, years	74.0 (68.0-81.0)	71.0 (62.0-79.0)	0.045
Male, n (%)	13 (31.7%)	123 (57.2%)	0.005
Weight, kg	83.2 (60.6-97.5)	83.2 (71.1-98.5)	0.278
Height, cm	163.0 (155.0-173.0)	168.0 (163.0-178.0)	0.001
**Comorbidities**			
Hypertension, n (%)	15 (36.6%)	110 (51.2%)	0.123
Diabetes mellitus, n (%)	10 (24.4%)	70 (32.6%)	0.395
Chronic kidney disease, n (%)	10 (24.4%)	43 (20.0%)	0.670
Heart failure, n (%)	20 (48.8%)	45 (20.9%)	<0.001
COPD, n (%)	10 (24.4%)	25 (11.6%)	0.053
Acute kidney injury, n (%)	33 (80.5%)	83 (38.6%)	<0.001
Charlson Comorbidity Index	7.0 (5.0-8.0)	5.0 (3.0-6.5)	<0.001
**Severity scores**			
SOFA score	8.0 (6.0-10.0)	5.0 (4.0-7.0)	<0.001
APACHE II score	25.0 (20.0-29.0)	20.0 (16.0-23.0)	<0.001
APS III score	68.0 (51.0-75.0)	39.0 (31.0-52.0)	<0.001
SAPS II score	51.0 (44.0-58.0)	36.0 (31.0-46.0)	<0.001
OASIS score	39.0 (33.0-47.0)	32.0 (26.0-37.0)	<0.001
SIRS score	3.0 (3.0-4.0)	3.0 (2.0-3.0)	0.006
Glasgow Coma Scale	15.0 (14.0-15.0)	15.0 (14.0-15.0)	0.215
**Laboratory values**			

Albumin, g/dL	2.6 (2.2-3.0)	3.1 (2.7-3.3)	<0.001
Hemoglobin, g/dL	8.6 (8.0-9.7)	9.2 (7.8-10.4)	0.284
Platelet, 10^3/uL	169.0 (103.0-306.0)	154.0 (116.5-208.0)	0.518
WBC, 10^3/uL	16.4 (10.5-21.7)	12.6 (8.8-17.9)	0.028
Lymphocytes, 10^3/uL	0.7 (0.5-1.6)	1.5 (0.9-2.3)	<0.001
Neutrophils, 10^3/uL	13.8 (9.6-18.1)	9.2 (6.3-14.3)	0.002
Creatinine, mg/dL	1.3 (1.0-1.8)	0.9 (0.7-1.3)	0.001
Lactate, mmol/L	2.2 (1.5-5.0)	1.8 (1.3-2.7)	0.016
BUN, mg/dL	32.0 (24.0-65.0)	17.0 (12.5-25.5)	<0.001
**Interventions**			
Mechanical ventilation, n (%)	36 (87.8%)	206 (95.8%)	0.091
Vasopressor use, n (%)	39 (95.1%)	179 (83.3%)	0.055
Antibiotic use, n (%)	40 (97.6%)	195 (90.7%)	0.215
**Nutrition-inflammation indices**			
mNUTRIC score	6.0 (4.0-7.0)	4.0 (3.0-5.0)	<0.001
PNI	31.0 (28.7-34.9)	38.6 (34.0-43.1)	<0.001
GNRI	80.4 (73.0-86.4)	86.4 (81.9-90.8)	<0.001
HALP	117.5 (55.3-208.3)	258.0 (135.6-462.3)	<0.001
mNUTRIC high risk, n (%)	38 (92.7%)	139 (64.7%)	<0.001
PNI <45 (low), n (%)	38 (92.7%)	184 (85.6%)	0.316
GNRI <98 (at risk), n (%)	41 (100.0%)	205 (95.3%)	0.372
INA grade 3-4 (high risk), n (%)	39 (95.1%)	197 (91.6%)	0.750
HALP T1 (lowest tertile), n (%)	27 (65.9%)	66 (30.7%)	<0.001

Note: *Baseline characteristics of the external validation cohort (Affiliated Hospital of Qingdao University, n=256), comparing non-survivors (n=41) with survivors (n=215). Continuous variables are median (IQR) compared by Mann-Whitney U test; categorical variables are n (%) compared by chi-squared or Fisher's exact test. Abbreviations as in Table 1.*

Table S2: Feature Selection Results

Feature	LASSO Coef.	LASSO Selected	Boruta Status	Final Selected
Age	0.1116	Yes	Confirmed	Yes

Acute kidney injury	0.2603	Yes	Confirmed	Yes
APS III score	0.3573	Yes	Confirmed	Yes
Charlson Comorbidity Index	0.3452	Yes	Confirmed	Yes
Albumin	-0.1364	Yes	Confirmed	Yes
Chloride	-0.2276	Yes	Confirmed	Yes
Fibrinogen	0.0587	Yes	Confirmed	Yes
Glucose	-0.0227	Yes	Confirmed	Yes
Lymphocyte %	-0.2518	Yes	Confirmed	Yes
PO2	-0.5282	Yes	Confirmed	Yes
RDW	0.3616	Yes	Confirmed	Yes
Red blood cells	-0.0048	Yes	Confirmed	Yes
SpO2	-0.0241	Yes	Confirmed	Yes
mNUTRIC high risk	0.0439	Yes	Confirmed	Yes
GNRI	-	No	Confirmed	
HALP	-	No	Confirmed	
HALP T1	-	No	Confirmed	
PNI	-	No	Confirmed	
Antibiotic use	0.0939	Yes	Rejected	
APACHE II score	-	No	Confirmed	
Chronic kidney disease	-0.0075	Yes	Rejected	
COPD	0.0262	Yes	Rejected	
Diabetes	-0.2540	Yes	Rejected	
Absolute lymphocyte count	-	No	Confirmed	
Anion gap	-0.0662	Yes	Rejected	
Creatinine	-	No	Confirmed	
Heart rate	-	No	Confirmed	
Neutrophil %	-	No	Confirmed	
Phosphate	0.0281	Yes	Rejected	

Potassium	-0.0417	Yes	Rejected	
PT	-	No	Confirmed	
BUN	-	No	Confirmed	
Ischemic heart disease	0.0025	Yes	Rejected	
OASIS score	-	No	Confirmed	
SAPS II score	-	No	Confirmed	
SIRS	0.0033	Yes	Rejected	
SOFA score	-	No	Confirmed	
Vasopressor use	0.2813	Yes	Rejected	

Note: *Results of LASSO (L1-penalized logistic regression) and Boruta feature selection from 71 candidate features shared between the two cohorts. "Final Selected" indicates features retained in the intersection of both methods (14 features). ICU length of stay was excluded a priori to prevent outcome leakage. '–' denotes a feature not assigned a LASSO coefficient (not selected by LASSO).*

Table S3: SHAP feature importance for the final LightGBM model in the derivation and external cohorts, compared with the published random-forest analysis

Feature	RF mean |SHAP| (dev)	Rank	LightGBM mean |SHAP| (dev)	Rank	LightGBM mean |SHAP| (ext)	Rank	Direction (LightGBM)
APS III	0.034	2	1.553	1	1.520	1	higher → higher risk
AKI	0.016	5	1.186	2	1.165	2	higher → higher risk
PO2	0.035	1	1.166	3	1.150	3	higher → lower risk
SpO2	0.011	11	1.035	4	1.107	4	higher → lower risk
Charlson Comorbidity Index	0.016	6	0.993	5	0.983	6	higher → higher risk
RDW	0.023	4	0.975	6	1.002	5	higher → higher risk
Lymphocyte percentage	0.034	3	0.836	7	0.850	8	higher → lower risk
Age	0.011	12	0.800	8	0.889	7	higher → higher

Feature	RF mean |SHAP| (dev)	Rank	LightGBM mean |SHAP| (dev)	Rank	LightGBM mean |SHAP| (ext)	Rank	Direction (LightGBM)
							risk
Chloride	0.012	9	0.726	9	0.704	9	higher → lower risk
Glucose	0.009	13	0.527	10	0.530	10	higher → lower risk
mNUTRIC high risk	0.003	14	0.520	11	0.457	12	higher → higher risk
Albumin	0.015	7	0.520	12	0.487	11	higher → lower risk
Red blood cell count	0.012	10	0.428	13	0.431	13	higher → lower risk
Fibrinogen	0.012	8	0.393	14	0.383	14	higher → higher risk

Note: SHAP values were computed with TreeExplainer. The LightGBM model is the final published model (300 trees, learning rate 0.05, 31 leaves; SMOTE applied to the derivation training data); the random forest (500 trees, max depth 8, no SMOTE) is the model used for interpretation in the original manuscript. Rank concordance: random forest vs. LightGBM in the derivation cohort, Spearman $\rho = 0.587$ ($p = 0.027$); LightGBM derivation vs. external, $\rho = 0.987$ ($p < 0.001$). Direction indicates the sign of the association between the feature value and the predicted probability of 28-day ICU mortality in the derivation cohort. PO2 = arterial oxygen tension; SpO2 = peripheral oxygen saturation; RDW = red cell distribution width; AKI = acute kidney injury.

Table S4: Missingness of the variables presented in the baseline table (Table 1) in the derivation and external validation cohorts

Variable	Derivation cohort (n = 917), n (%)	External validation cohort (n = 256), n (%)
Age, years	0 (0.0)	0 (0.0)
Male, n (%)	0 (0.0)	0 (0.0)
Weight, kg	0 (0.0)	0 (0.0)
Height, cm	0 (0.0)	0 (0.0)
Hypertension, n (%)	0 (0.0)	0 (0.0)
Diabetes mellitus, n (%)	0 (0.0)	0 (0.0)

Variable	Derivation cohort (n = 917), n (%)	External validation cohort (n = 256), n (%)
Chronic kidney disease, n (%)	0 (0.0)	0 (0.0)
Heart failure, n (%)	0 (0.0)	0 (0.0)
COPD, n (%)	0 (0.0)	0 (0.0)
Acute kidney injury, n (%)	0 (0.0)	0 (0.0)
Charlson Comorbidity Index	0 (0.0)	0 (0.0)
SOFA score	0 (0.0)	0 (0.0)
APACHE II score	0 (0.0)	0 (0.0)
APS III score	0 (0.0)	0 (0.0)
SAPS II score	0 (0.0)	0 (0.0)
OASIS score	0 (0.0)	0 (0.0)
SIRS score	0 (0.0)	0 (0.0)
Glasgow Coma Scale	0 (0.0)	0 (0.0)
Albumin, g/dL	0 (0.0)	0 (0.0)
Hemoglobin, g/dL	0 (0.0)	0 (0.0)
Platelet, 10^3/uL	0 (0.0)	0 (0.0)
WBC, 10^3/uL	3 (0.3)	0 (0.0)
Lymphocytes, 10^3/uL	0 (0.0)	0 (0.0)
Neutrophils, 10^3/uL	0 (0.0)	0 (0.0)
Creatinine, mg/dL	0 (0.0)	0 (0.0)
Lactate, mmol/L	38 (4.1)	0 (0.0)
BUN, mg/dL	0 (0.0)	0 (0.0)
Mechanical ventilation, n (%)	0 (0.0)	0 (0.0)
Vasopressor use, n (%)	0 (0.0)	0 (0.0)
Antibiotic use, n (%)	0 (0.0)	0 (0.0)
Fibrinogen	267 (29.1)	70 (27.3)

Note: Values are the number (percentage) of patients with missing values. Derivation-cohort counts for WBC and lactate (3 and 38 patients) were derived from the reported percentages. Among all 48 extracted continuous variables, missingness ranged from 0.1% to 29.1% and was below the pre-specified 30% threshold for every variable, so no variable was excluded from the analysis. Fibrinogen is not presented in the baseline table (Table 1); it enters the analysis only as one of the 14 features of the machine-learning model. All components of the five nutrition–inflammation indices (albumin, lymphocyte count, haemoglobin, platelet count, body weight, height) and all covariates in the regression models had 0% missingness in both cohorts.

Table S5: Variance inflation factors (VIF) for the covariates of the fully adjusted model (Model 3)

Covariate (Model 3)	VIF
Age	1.37
Male sex	1.01
APACHE II	1.89
SOFA	1.82
Acute kidney injury	1.27
Charlson Comorbidity Index	1.59
Mechanical ventilation	1.12
Vasopressor use	1.19

Note: VIF values were computed on the design matrix of the fully adjusted model (Model 3) in the derivation cohort (n = 917). The conventional threshold of VIF ≥ 5 was applied to define problematic multicollinearity; all values were ≤ 1.90. VIFs were essentially unchanged when the exposure variable (e.g., HALP lowest tertile) was included in the design matrix (maximum VIF 1.90). APACHE II = Acute Physiology and Chronic Health Evaluation II; SOFA = Sequential Organ Failure Assessment; AKI = acute kidney injury; CCI = Charlson Comorbidity Index.

Table S6: Box–Tidwell tests of the linearity-in-the-logit assumption for continuous covariates

Covariate	Minimum	Shift applied	x·ln(x) p-value
Age	24	+0	0.609
APACHE II	4	+0	0.708
SOFA	0	+1	0.401
Charlson Comorbidity Index	0	+1	0.219

Note: For each continuous covariate, an x·ln(x) interaction term was added to the fully adjusted logistic model; a non-significant interaction (p > 0.05) indicates that the linearity-in-the-logit assumption is satisfied. For covariates with a minimum value of 0 (SOFA, CCI), a shift of +1 was applied before the logarithmic transformation.

Table S7: Component-aware and sensitivity logistic regression models for the association of each nutrition–inflammation index with 28-day ICU mortality

Index	Model	OR (95% CI)	p-value
**mNUTRIC ≥5**			
	Model 3 (original)	1.72 (0.77–3.83)	0.188
	Model 3a (without SOFA)	1.82 (0.82–4.05)	0.141
	Model 3b (without APACHE II)	2.10 (0.98–4.50)	0.057

Index	Model	OR (95% CI)	p-value
	Model 3c (without AKI)	1.70 (0.77–3.76)	0.190
	Model 3d (without SOFA & AKI)	1.86 (0.85–4.08)	0.123
	Component-aware (sex, AKI, ventilation, vasopressor only)	3.93 (1.99–7.78)	<0.001
**PNI <45**			
	Model 3 (original)	2.48 (1.09–5.66)	0.031
	Model 3a (without SOFA)	2.58 (1.13–5.89)	0.024
	Model 3b (without APACHE II)	2.42 (1.07–5.50)	0.034
	Model 3c (without AKI)	2.57 (1.13–5.86)	0.025
	Model 3d (without SOFA & AKI)	2.74 (1.20–6.25)	0.017
**GNRI <98**			
	Model 3 (original)	2.18 (0.49–9.73)	0.308
	Model 3a (without SOFA)	2.27 (0.51–10.12)	0.284
	Model 3b (without APACHE II)	2.25 (0.51–9.98)	0.287
	Model 3c (without AKI)	1.84 (0.42–8.01)	0.416
	Model 3d (without SOFA & AKI)	1.91 (0.44–8.27)	0.389
**INA grade 3–4**			
	Model 3 (original)	1.21 (0.48–3.02)	0.684
	Model 3a (without SOFA)	1.26 (0.50–3.14)	0.624
	Model 3b (without APACHE II)	1.26 (0.51–3.14)	0.613
	Model 3c (without AKI)	1.07 (0.44–2.61)	0.889
	Model 3d (without SOFA & AKI)	1.11 (0.45–2.71)	0.821
**HALP T1**			
	Model 3 (original)	3.41 (2.13–5.46)	<0.001
	Model 3a (without SOFA)	3.32 (2.08–5.29)	<0.001
	Model 3b (without APACHE II)	3.50 (2.19–5.58)	<0.001
	Model 3c (without AKI)	3.94 (2.48–6.27)	<0.001
	Model 3d (without SOFA & AKI)	3.86 (2.43–6.12)	<0.001

Note: Model 3 (original): adjusted for age, sex, APACHE II, SOFA, AKI, Charlson Comorbidity Index, mechanical ventilation, and vasopressor use. Model 3a: without SOFA. Model 3b: without APACHE II. Model 3c: without AKI. Model 3d: without SOFA and AKI. The component-aware model for mNUTRIC adjusts only for covariates not embedded in the mNUTRIC calculation (sex, AKI, mechanical ventilation, and vasopressor use), because age, APACHE II, SOFA, and comorbidity count are direct components of the score. OR = odds ratio; CI = confidence interval.

Table S8: Fully nested cross-validation with feature selection repeated inside each training fold

Outer fold	Features, n	CV AUROC	External AUROC
1	9	0.848	0.944
2	8	0.821	0.960
3	11	0.849	0.955
4	6	0.824	0.914
5	9	0.803	0.955
Published pipeline (selection on full cohort)	*14*	*0.854*	*0.956*

Note: In each outer fold, LASSO (inner-cross-validated regularization strength) and Boruta (150 iterations) were refit on the training fold only, and their intersection was used to train LightGBM (published hyperparameters; SMOTE within the training fold). "CV AUROC" is evaluated on the untouched held-out fold; "External AUROC" is evaluated on the independent external cohort (n = 256). The published pipeline row repeats the original analysis (selection on the full derivation cohort) for reference. The re-implemented selection procedure recovered 7 of the 14 published features on the full cohort (the original analysis was performed in R with mice-imputed data, so exact replication of the selected set is not expected); the nested analysis is therefore a conservative stress-test of the pipeline rather than an exact reproduction. CV = cross-validation.

Table S9: Harmonized mNUTRIC analysis using the identical 4-item definition (high-risk ≥4) in both cohorts

Cohort and mNUTRIC definition	High risk, %	AUROC (95% CI)	Model 3 OR (95% CI)	p-value
Derivation, 5-item mNUTRIC ≥ 5 (published)	66.6	0.734 (0.685–0.778)	1.72 (0.77–3.83)	0.188
Derivation, 4-item mNUTRIC ≥ 4 (harmonized)	66.7	0.733 (0.685–0.777)	1.67 (0.75–3.73)	0.212
External, 4-item mNUTRIC ≥ 4 (published)	69.9	0.742 (0.655–0.819)	1.54 (0.35–6.84)	0.569
LightGBM external AUROC with harmonized mNUTRIC_hi feature	–	0.957	–	–

Note: AUROC values are for the continuous mNUTRIC score with 95% confidence intervals from 1,000 stratified bootstrap iterations; odds ratios are from the fully adjusted (Model 3) logistic regression for the binary high-risk classification. Values are from the present re-analysis; the corresponding AUROCs reported in Table 3 of the manuscript are 0.723 (derivation, 5-item) and 0.731 (external, 4-item), with minor numerical differences reflecting reconstruction of

the score from the raw components. The last row reports the external-validation AUROC of the LightGBM model retrained with the harmonized binary mNUTRIC feature. OR = odds ratio; CI = confidence interval.
